# Per- and Poly-Fluoroalkyl Substances, and Organophosphate Flame Retardants in the Upper Yangtze River: Occurrence, Spatiotemporal Distribution, and Risk Assessment

**DOI:** 10.3390/toxics13020116

**Published:** 2025-02-01

**Authors:** Wen Sun, Zhiyou Fu, Yueyue Liu, Yingchen Bai, Yuyan Zhao, Chen Wang, Fengchang Wu

**Affiliations:** 1 College of Geo-Exploration Science and Technology, Jilin University, Changchun 130026, China; sunwen0039@163.com (W.S.); zhaoyuyan@jlu.edu.cn (Y.Z.); 2 State Key Laboratory of Environmental Criteria and Risk Assessment, Chinese Research Academy of Environmental Sciences, Beijing 100012, China; fuzy@craes.org.cn (Z.F.); liuyueyue0103@mail.bnu.edu.cn (Y.L.); baiyc@craes.org.cn (Y.B.)

**Keywords:** Contaminants of Emerging Concern (CECs), surface water, spatiotemporal distribution, risk assessment

## Abstract

Contaminants of Emerging Concern (CECs), including per- and polyfluoroalkyl substances (PFASs) and organophosphate flame retardants (OPFRs), have raised global concerns due to their persistence, bioaccumulation potential, and toxicity. This study presents a comprehensive investigation of the occurrence, spatiotemporal distribution, potential sources, and the ecological and human health risks associated with 18 PFASs and 9 OPFRs in the surface waters of the upper Yangtze River, China. The water samples were collected from the main stream and five major tributaries (Min, Jinsha, Tuo, Jialing, and Wu Rivers) in 2022 and 2023. The total concentration of PFASs and OPFRs ranged from 16.07 to 927.19 ng/L, and 17.36 to 190.42 ng/L, respectively, with a consistently higher concentration observed in the main stream compared to the tributaries. Ultra-short-chain PFASs (e.g., TFMS) and halogenated OPFRs (e.g., TCPP) were the predominant compounds, likely originating from industrial discharges, wastewater effluents, and other anthropogenic sources. Ecological risk assessments indicated low-to-moderate risks at most sampling sites, with higher risks near wastewater discharge points. Human health risk evaluations suggested negligible non-carcinogenic risks but identified potential carcinogenic risks from OPFR exposure for adults at specific locations, particularly in Leshan city. This study highlights the importance of understanding the fate and impacts of PFASs and OPFRs in the upper Yangtze River, and provides valuable insights for developing targeted pollution control strategies and risk management measures.

## 1. Introduction

Contaminants of Emerging Concern (CECs), encompassing a wide array of anthropogenic compounds such as pharmaceuticals, personal care products, and endocrine-disrupting chemicals, have garnered significant attention in recent years due to their ubiquitous presence in the environment and potential adverse effects on ecosystems and human health [1,2]. Among these, per- and polyfluoroalkyl substances (PFASs) and organophosphate flame retardants (OPFRs) have emerged as two important classes, drawing particular attention due to their unique properties and widespread use [3,4]. PFASs are used in a variety of consumer products (e.g., food packaging, surfactants, pesticides, and non-stick coatings) due to their unique chemical properties (e.g., surface activity, acid resistance, chemical stability, heat resistance, water and oil repellency) [5,6,7]. Additionally, PFASs are often found in the environment because their chemical structure makes them highly resistant to degradation through processes like hydrolysis, photolysis, and biodegradation [8,9,10]. As an alternative to bromine-based flame retardants, OPFRs have gained widespread adoption as flame retardants and plasticizers in various industrial products [11,12]. However, unlike their brominated counterparts, OPFRs do not chemically polymerize with the substrates to which they are applied, which facilitates the release of OPFRs into the environment during the production, use, and disposal of treated products [12,13]. The widespread use of PFASs and OPFRs, coupled with their continuous emissions and the inadequacy of current wastewater treatment technologies, has resulted in the ubiquitous presence of these contaminants in surface waters worldwide [14,15,16,17,18,19]. This highlights the need for a comprehensive understanding of the occurrence, fate, transport, and effects of these contaminants in aquatic environments, as well as the development of effective strategies for their mitigation and remediation to protect ecosystem and human health [20,21].

The ubiquitous presence of PFASs and OPFRs in aquatic environments, coupled with growing evidence of their adverse effects on organisms, has raised growing significant concerns. Studies have linked PFAS exposure to a range of health issues, including immune dysfunction, metabolic disorders, reproductive toxicity, and an increased risk of various cancers [22]. In response to these findings, international agencies such as the United States Environmental Protection Agency (US EPA) and the European Commission have implemented restrictions on the use of PFASs to mitigate their environmental and human health impacts [23]. Similarly, OPFRs have raised significant concerns due to their structural similarities with organophosphate pesticides, which are known for their multiple toxicities, including neurotoxicity [24]. Research has associated OPFRs with various negative health outcomes, such as genotoxicity, endocrine disruption, immunotoxicity, neurotoxicity, and potential carcinogenicity [25,26]. Reflecting these concerns, the European Union has taken proactive measures by banning several OPFRs in children’s products due to their potential carcinogenic risks [27]. Given the growing evidence highlighting the adverse effects of PFASs and OPFRs, the long-term monitoring of these CECs in aquatic environments is crucial for understanding their environmental fate, transport, and impacts, as well as for informing the development of effective regulatory measures and remediation strategies to safeguard ecosystem and human health.

The Yangtze River Basin, the largest river basin in Eurasia, plays a vital role in supporting the livelihoods of hundreds of millions of people in China and is one of the country’s most economically developed regions [28]. However, in recent decades, the basin, particularly its upper reaches, has experienced significant pollution [29]. While several studies have investigated the occurrence and concentration of PFASs and OPFRs in Yangtze River [30,31,32,33], most have focused on the middle and lower reaches, leaving a critical knowledge gap regarding the presence and impacts of these contaminants in the surface water of the upper reaches. Chongqing and Sichuan, two key provincial regions in the upper Yangtze River, host rapidly developing industries and are major consumers of chemicals and pharmaceuticals. These activities likely contribute to heightened pollution from PFASs and OPFRs in the basin, further emphasizing the need for a comprehensive understanding of their environmental fate and impacts. Given the growing concerns about the adverse effects of PFASs and OPFRs on ecosystem and human health, and the limited information available on their occurrence in the upper Yangtze River, it is crucial to evaluate the pollution status and conduct a comprehensive risk assessment of these contaminants in this region.

To address the knowledge gaps and growing concerns surrounding PFASs and OPFRs in the upper Yangtze River, this study systematically investigated the occurrence, spatiotemporal distribution, and potential sources of 18 PFASs and 9 OPFRs in water bodies of the region, including the main stream and four tributaries. By employing advanced analytical techniques and comprehensive sampling strategies, this research aimed to provide a deep understanding of the environmental behavior and fate of these contaminants in this ecologically and economically important region. Furthermore, a comprehensive risk assessment was conducted to evaluate the potential ecological and human health risks associated with PFASs and OPFRs in the upper Yangtze River. The findings of this study contribute to the development of a robust risk assessment framework for these contaminants in China’s water environment, serving as a scientific foundation for informed decision-making and effective pollution control strategies to safeguard the environment and human health in Yangtze River.

## 2. Materials and Methods

### 2.1. Chemicals and Reagents

A total of 18 PFASs and 9 OPFRs were monitored in this study. The details of the target compounds, including their acronyms, CAS numbers, empirical formulas, dissociation constants(pKa), Log K_ow_ values, and common uses, are provided in Supplementary Materials (Text S1, Table S1). Mass-labeled compounds containing ^2^H and ^13^C isotopes were used as the internal standards (IS). All standards were purchased from J&K Scientific Ltd. (Beijing, China) and Sigma-Aldrich (St. Louis, MO, USA). Ultrapure water was obtained from a Milli-Q water purification system (Millipore, Bedford, MA, USA). HPLC-MS-grade methanol, acetonitrile, ammonia, and dichloromethane were purchased from Fisher Chemical, Inc. (Waltham, MA, USA). LC-MS-grade ammonium acetate and formic acid (≥95%) were purchased from Sigma-Aldrich Inc. (St. Louis, MO, USA). Solid-phase extraction (SPE) cartridges Oasis HLB (500 mg, 6 cc) and Oasis WAX (500 mg, 6 cc) were purchased from Waters Inc. (Milford, MA, USA).

### 2.2. Study Area and Sample Collection

**Figure 1:**
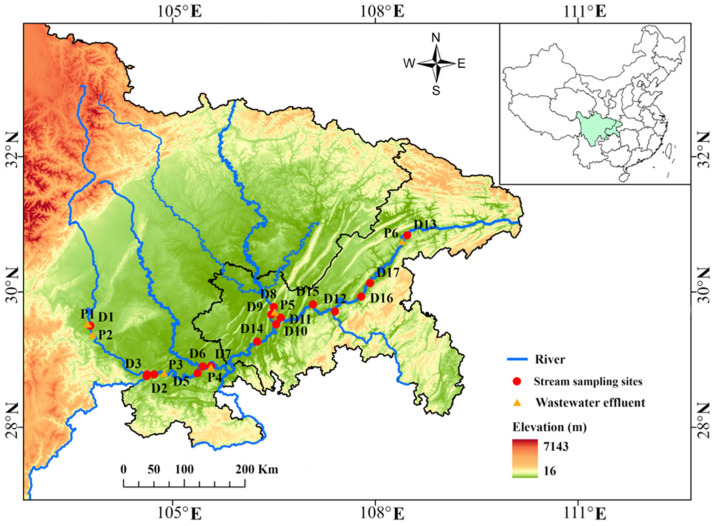
Sampling locations in the Sichuan and Chongqing Reach of Yangtze River. Red dots represent stream sampling sites, and yellow triangles represent wastewater effluent. There were 13 surface water sampling sites (D1–D13) along 2022, and 17 surface water sampling sites (D1–D17) along the 2023.

The Sichuan and Chongqing reaches of Yangtze River were selected as the study area. Water samples were collected from both the main channel of Yangtze River and its key tributaries, including the Min River, Jinsha River, Tuo River, Jialing River, and Wu River. Water samples were collected from the upper Yangtze River during two sampling campaigns in August 2022 (dry season) and August 2023 (flood season). In August 2022, a total of 19 samples were collected, including 13 samples from stream sites and 6 samples from wastewater treatment plant (WWTP) effluent sites. The sampling campaign was expanded in August 2023, with a total of 23 samples collected, comprising 17 stream samples and 6 WWTP effluent samples (Figure 1). For each sampling point, three individual samples (*n* = 3) were combined and thoroughly mixed to prepare a representative sample. Detailed information on the sampling locations is provided in Table S2. The samples were collected in amber glass bottles (4 L) that were pre-cleaned and rinsed with tap water, methanol, Milli-Q water, and water at the sampling site. The samples were stored and transported at 4 °C protected from light, and extracted within 24 h.

DZB-718L portable multiparameter water quality meter (Leici Inc., Shanghai, China) was used to measure water temperature (T), pH, oxidation–reduction potential (ORP), conductivity (Cond), dissolved oxygen (DO) at each site. The water quality parameters are summarized in Table S2.

### 2.3. Sample Preparation, Extraction, and Analysis

Samples (500 mL each) were filtered through 0.45 μm glass microfiber filters (Whatman GF/A) and stored at −20 °C until further processing and analysis. Targeted analyses were conducted using the methods described in previous studies [34,35,36]. Briefly, the filtered water samples were spiked with 50 ng of mixed internal standards and then enriched using activated SPE cartridges, and eluted with an appropriate solvent. The eluates were then concentrated under a stream of nitrogen (N_2_), and reconstituted in methanol to a final volume of 500 μL (detailed in Text S2).

The target analytes were quantified using an ultra-high-performance liquid chromatography system (HPLC, 1290, Agilent, Santa Clara, CA, USA) coupled to a triple quadrupole mass spectrometer (MS/MS, 6460A, Agilent, Santa Clara, CA, USA) equipped with an electrospray ionization (ESI) interface (Agilent Technologies, Santa Clara, CA, USA). Data analysis, both qualitative and quantitative, was performed using MassHunter workstation software (GL Sciences, Tokyo, Japan). The injection volume for each sample was 5 μL. Detailed instrumental parameters and analytical methods are provided in Table S3.

### 2.4. Quality Assurance and Quality Control

Isotopically labeled internal standards and matrix-matched calibration were used to quantify target compounds [37]. The calibration curves demonstrated excellent linearity, with correlation coefficients (R^2^) ≥ 0.99 for the concentration ranges of 1–200 ng/mL for PFASs and 1–500 ng/mL for OPFRs. The limits of detection (LOD) and quantification (LOQ) were determined based on the signal/noise ratios of 3 and 10, respectively. Each group (3–5 samples) contained a blank sample of which the concentration was always below the LOD. Detailed information on LOD, LOQ, and the recoveries of target compounds is shown in Table S4.

### 2.5. Annual Flux Estimation

The annual emission flux of target pollutants in the upper reaches of Yangtze River is estimated by the following formula [38]:

(1) $$F=C\times Q\times 10^{-12}$$

where the flux is expressed as F (tons/year), C is the concentration of pollutants in the water phase (ng/L), and Q is the annual runoff of the basin. The contribution of tributaries to the main stream of Yangtze River can be estimated. The annual average flow data of the upper Yangtze River main stream and its tributaries are from the Statistical Yearbook (Table S5).

### 2.6. Ecological Risk Assessment

The ecological risk assessment was based on the European Commission Guidelines (EC, 2003) and recent research methods [39], and risk quotient (RQ) value was estimated by the following formula:

(2) $$RQ=\frac{MEC}{PNEC}$$

where, MEC refers to measured environmental concentration and PNEC refers to the predicted no-effect concentration of the analyte.

The ecological risk of the mixtures was assessed using the following formula [40]:

(3) $${RQ}_{STU}=\sum_{i}^{n}\frac{{MEC}_{i}}{{min({{PNEC}_{i,algae},PNEC}_{i,crustacean},PNEC}_{i,fish})}$$

where STU refers to the sum of Toxic Units, and MEC_i_ is the measured environmental concentration of the *i*th component in an n-compound mixture.

Details of PNECs and RQs are provided in Table S6. Preliminary risk assessment level of pollutants: no risk (≤0.01), low risk (0.01–0.1), medium risk (0.1–1), or high risk (>1) [41].

### 2.7. Human Health Risk Assessment

Health risk assessments for human exposure to chemicals typically include both non-carcinogenicity (non-CR) and carcinogenicity (CR) risk [31]. The hazard index (HI) is usually used to assess the non-CR of a compound. The average daily intake (ADI) of PFASs and OPFRs through drinking water was estimated and compared with the reference dose (RfD) and slope factors (SFs) to derive the HI and CR values. The total HI and CR were estimated by summing the studied compounds species HI and CR [31,42]. ADI, HI, and CR were calculated using the following formula:

(4) $${ADI}_{i}=\frac{C_{i}\times {IR}_{i}\times EF\times ED}{AT\times BW}$$

(5) $${HI}_{i}=\frac{{ADI}_{i}}{RfDi}$$

(6) $$HI=\sum_{i}^{n}{HI}_{i}$$

(7) $${CR}_{i}={ADI}_{i}\times {SF}_{i}$$

(8) $$CR=\sum_{i}^{n}{CR}_{i}$$

where C_i_ is the concentration of each PFAS and OPFR in water (ng/L), IR_i_ is the intake rate of water (L/day), EF is the exposure frequency (day/year), ED is the exposure duration (year), AT is the average time (days), and BW is the average body weight (kg). *RfD_i_* is the reference dose for each PFASs and OPFRs (ng/kg/day), and SF_i_ is the slope factors for each PFASs and OPFRs via ingestion.

The parameter values used in the health risk assessment are provided in Tables S7 and S8. Generally, HI < 1 is considered to be free of adverse health effects, whereas HI > 1 indicates the possibility of non-CR exceeding acceptable levels [43]. CR < 10^−6^ was considered negligible, and 10^−6^ < CR < 10^−4^ and CR > 10^−4^ indicated potential and high carcinogenic risks, respectively [44].

### 2.8. Statistical Analysis

SPSS 19.0 and Origin 2021 were used for data analysis. ArcGIS 10.7 was used for drawing the map of the sample sites and analyzing the spatial variation in PFAS and OPFR concentration in the upper Yangtze River. Nonmetric multidimensional scaling (NMDS) and heatmaps were produced with R statistical software (version 3.5.1). The Spearman correlation analysis was used to examine the possible correlations among various PFASs and OPFRs in the samples.

## 3. Results and Discussion

### 3.1. Occurrence and Concentration of PFASs and OPFRs in the Upper Yangtze River

**Figure 2:**
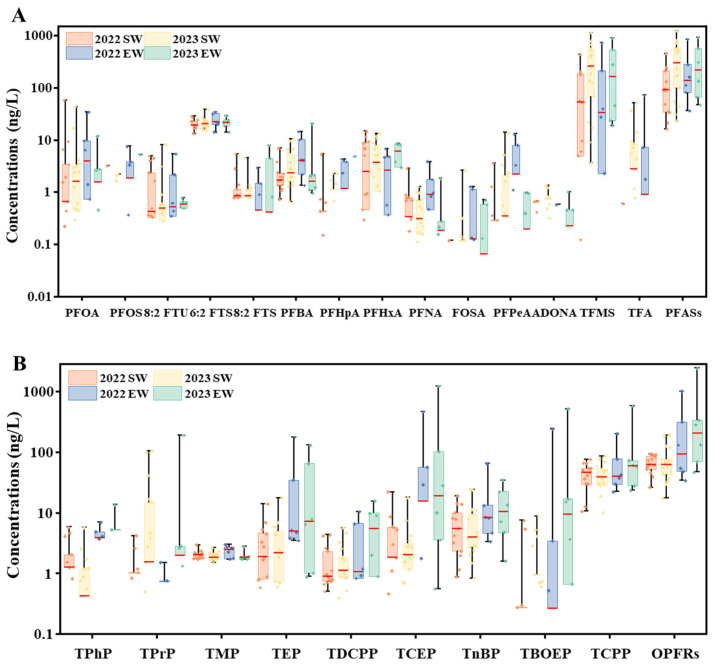
Concentrations of the PFASs (**A**) and OPFRs (**B**) in the upper Yangtze River in 2022 and 2023. SW: surface water, EW: effluent wastewater. Box charts (bottom to top) presented the 5th, 25th, 50th, 75th, and 95th percentile values. The absence of a box indicates that the detection rate of the compound is less than 50%.

The concentration of PFASs and OPFRs in surface water and effluent wastewater (minimum, maximum, median, mean, and detection frequency) are summarized in Table 1 and shown in Figure 2A,B. In surface water, the total PFAS concentration ranged from 16.07 to 454.68 ng/L in 2022, and from 23.13 to 1231.11 ng/L in 2023. The concentrations in 2023 were higher than those in 2022, both for individual compounds and for total PAFSs (Figure 2A). Among the 18 PFASs analyzed, 6:2 FTS and PFBA had the highest detection frequencies, with a detection frequency of 100%. The main PFASs were TFMS (2022: ND to 433.46 ng/L, 2023: 3.59 to 1119.56 ng/L) and 6:2 FTS (2022: 13.22 to 28.45 ng/L, 2023: 15.15 to 38.36 ng/L) (Figure 2A). These results are not entirely consistent with previous studies on aquatic PFASs in Yangtze River [33,45], which may be due to the different types of pollutants detected. Notably, TFMS was detected at a relatively high concentration, of ten reaching hundreds of ng/L, which has not been reported previously. TFMS, is a strong acid widely used as a catalyst in chemical reactions, is especially prevalent in the production of lithium-ion batteries [46,47]. The elevated TFMS concentration in surface waters are likely attributed to significant point source inputs [48], such as emissions from lithium ion battery manufacturers located in Sichuan and Chongqing [49]. As shown in Table S9, previous studies have also reported the presence of PFASs in surface waters worldwide. Overall, with the exception of TFMS (for which environmental data remain scarce), the total concentration of PFASs in the upper Yangtze River is higher than that in the Yellow River, Pearl River and Poyang Lake [14,45], and lower than that in Taihu Lake, the Thames River in the UK, and urban rivers in Europe and the United States [8,14,50]. In terms of individual PFAS, the concentration of PFOA (ND to 86.90 ng/L) and PFOS (0.36-12.12 ng/L) in this study were lower than those previously reported in Yangtze River surface water [29,34]. These differences may be due to the fact that PFOA and PFOS have been restricted in recent years [51]. PFBA, PFHxA, PFBS, and 6:2 FTS were used as substitutes for PFOA and PFOS, and their detection concentrations were higher than previously reported [14,33]. In this study, the total concentration of OPFRs in surface water ranged from 26.25 to 93.19 ng/L in 2022, and from 17.36 to 190.42 ng/L in 2023. The detection concentrations of the nine OPFRs included in the test were similar in 2022 and 2023 (Figure 2B). Furthermore, three alkyl-OPFRs (TMP, TEP, and TnBP) were found in more than 90% of the samples. Three halogenated OPFRs, TCEP, TCPP, and TDCPP, were found in more than 70% of the samples. TCPP was the main OPFR, with the concentration in the range from 10.65 to 75.57 ng/L in 2022 and in the range from ND to 86.19 ng/L in 2023, with detection frequencies > 95%. For aryl-OPEs, the detection rates of TPhP were 53% in 2022 and 62% in 2023, and the concentration ranged from ND to 5.84 ng/L in 2022 and ND to 6.96 ng/L in 2023. Generally, this study revealed that the contents of the OPEs in the water samples of the upper Yangtze River could be ranked as follows: halogenated OPFRs > alkyl-OPFRs > aryl-OPFRs. The production and application volume, as well as photodegradation may explain the different detection rates and the concentration of OPFRs [52,53]. These results were consistent with previous studies on OPFRs in Yangtze River [31,36]. The high detection rates (100%) of TMP in both 2022 and 2023 might be related to its high-water solubility (5.00 × 10^5^ mg/L) and frequent use in China, such as solvents and catalysts in organic synthesis, pharmaceutical intermediates, plasticizers, and flame retardants [54]. The dominance of TCPP in aquatic environments could be related to its relative persistence [55]. As listed in Table S10, the levels of the OPFRs in the upper Yangtze River were comparable to that in Xiangjiang River [56], higher than that in Brazilian surface water [57], but lower than those detected in rivers and lakes in North America, Europe, and other regions of China [31,36,58,59,60,61,62,63].

Regarding effluent wastewater, the total concentration of PFASs ranged from 36.41 to 849.82 ng/L in 2022, and from 47.45 to 926.93 ng/L in 2023 (Figure 2A). The most frequently detected PFASs in effluents were 6:2 FTS (100%), PFBA (100%), and TFMS (92%). The primary PFASs detected in these samples were TFMS:ND to 726.98 ng/L in 2022, and 18.61 to 888.25 ng/L in 2023. In fact, there were no significant differences between surface water and effluent wastewater samples, either in terms of the concentration of individual compounds or total PFASs. This may be due to the strong antioxidant properties of PFASs [35]. Furthermore, the high concentration of PFASs observed in effluent wastewater suggests that WWTPs are ineffective at removing PFASs [64]. Therefore, WWTPs could be one of the main sources of PFASs in surface water [65], and lead to a high concentration in water from the upper reaches of Yangtze River. For OPFRs, the total concentration of OPFRs ranged from 33.95 to 1007.79 ng/L in 2022, and from 46.83 to 2427.21 ng/L in 2023 (Figure 2B). The most frequently detected OPFRs were TMP, TEP, TnBP, and TCPP, reaching 100%. Additionally, the highest concentration for TPrP, TEP, TCEP, TBOEP, and TCPP in effluent water were 189.98, 176.51, 1221.10, 514.91, and 572.81 ng/L, respectively. In fact, the concentration of OPFRs in effluent wastewater was much higher than that in surface water (Figure 2B). This discrepancy may be attributed to the photolysis of OPFRs after prolonged exposure to sunlight in surface waters, as well as the dilution effect of larger water bodies [31,36]. The chemical compositions of effluent water are listed in Table S11.

### 3.2. Spatiotemporal Distribution of PFASs and OPFRs

**Figure 3:**
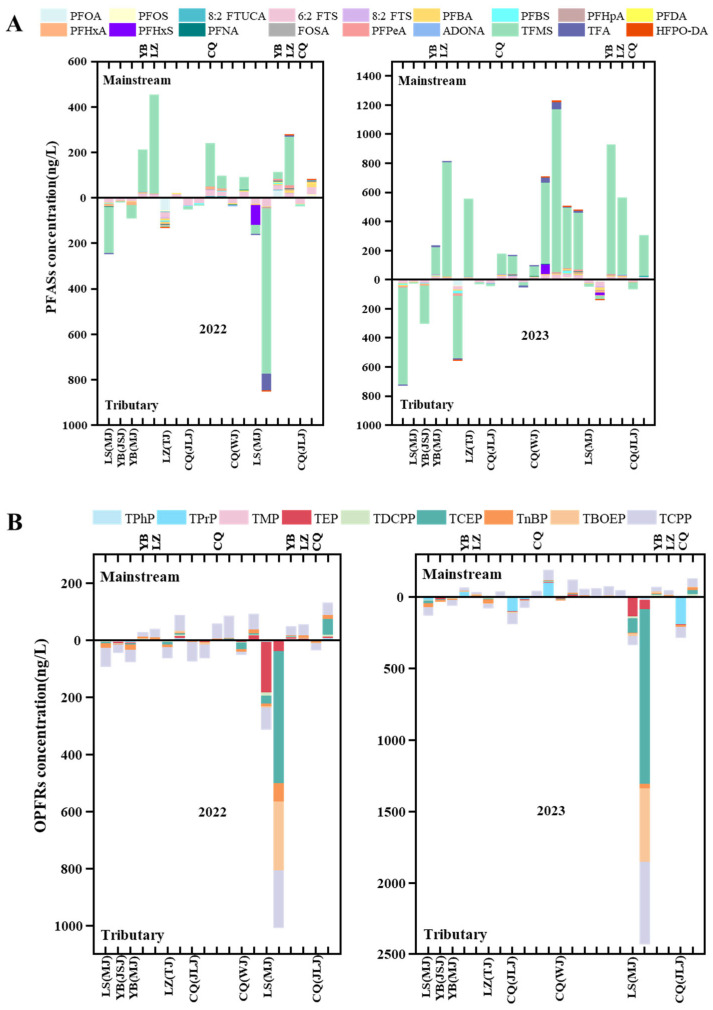
Total PFAS (**A**) and OPFR (**B**) concentrations (ng/L) in samples of Yangtze River in 2022 and 2023. For each figure, the upright bar refers to the main stream and the inverted bar refers to the tributaries.

The total mean concentration of PFASs in the main stream was higher than in the tributaries (main stream: 184.82 ng/L in 2022 and 496.84 ng/L in 2023; the tributaries: 85.46 ng/L in 2022 and 247.37 ng/L in 2023) (Figure 3A). Furthermore, the concentration of PFASs in different tributaries also varied and could be ranked as follows: Minjiang River (243.99 ng/L in 2022, 721.88 ng/L in 2023) > Tuojiang River (130.46 ng/L in 2022, 554.56 ng/L in 2023) > Jialing River (48.97 ng/L in 2022, 44.48 ng/L in 2023) > Wujiang River (33.78 ng/L in 2022, 53.88 ng/L in 2023) > Jinsha River (16.07 ng/L in 2022, 23.13 ng/L in 2023) (Figure 3A). These reflected the stability of the contribution of various tributaries in the upper reaches of Yangtze River to the main-stream PFASs in 2022 and 2023. The levels of PFASs in Jialing River, Wujiang River, and Jinsha River decreased significantly, which may relate to the sites far away from the pollution sources of urban industrial areas. From a spatial distribution perspective, the highest PFAS concentration was observed at the sampling points in the main stream in Luzhou (453.56 ng/L in 2022 and 814.18 ng/L in 2023) and Chongqing (239.06 ng/L in 2022 and 1231.11 ng/L in 2023). The degree of PFAS pollution is directly related to the population density and industrial structure of the surveyed regions [66]. These elevated concentrations may be attributed to point-source inputs from upstream fluorine chemical industrial parks located near Luzhou and Chongqing [34]. The total average concentration of OPFRs in the main stream and tributaries was similar in 2022 and 2023 (main stream: 65.62 ng/L in 2022, 76.35 ng/L in 2023 and tributaries: 66.34 in 2022, 80.92 ng/L in 2023) (Figure 3B). The concentration of OPFRs in different tributaries also varied, and their ranking in 2022 and 2023 was not entirely consistent (Figure 3B). In 2022, the tributary OPFRs levels were ranked as follows: Minjiang River (93.19 ng/L) > Jialing River (73.35 ng/L) > Tuojiang River (62.90 ng/L) > Wujiang River (52.01 ng/L) > Jinsha River (45.08 ng/L). In 2023, the ranking was Jialing River (186.95 ng/L) > Minjiang River (125.73 ng/L) > Tuojiang River (74.90 ng/L) > Jinsha River (30.11 ng/L) > Wujiang River (17.36 ng/L). High concentrations of OPFRs were detected in water bodies in Leshan and Chongqing. The population density and human activities were the important reason for the changes in the concentration of OPFRs on the spatial scale [30,55,67]. Chongqing, a municipality with a dense population and a highly developed economy, exhibited high OPFR levels. Additionally, the incomplete removal of halogenated OPFRs in wastewater by WWTPs was one of the important sources of OPFR pollution in rivers [14]. For example, high concentrations of OPFRs, especially TEP (176.51 ng/L), TCEP (1221.10 ng/L), TCPP (514.91 ng/L), and TBOEP (1221.10 ng/L), were detected in the effluents of Yibin WWTPs. The concentration of OPFRs was influenced by anthropogenic activities such as vehicle emissions, industrial effluents, and release from daily household products which may be the main sources of OPFRs. This study found that the concentration of OPFRs in urban areas and industrial areas with active human activities was higher than that in residential areas with low population density. It was reported that the release of building materials, vehicle emissions, industrial wastewater, etc. may be the main sources of OPFRs [56].

**Figure 4:**
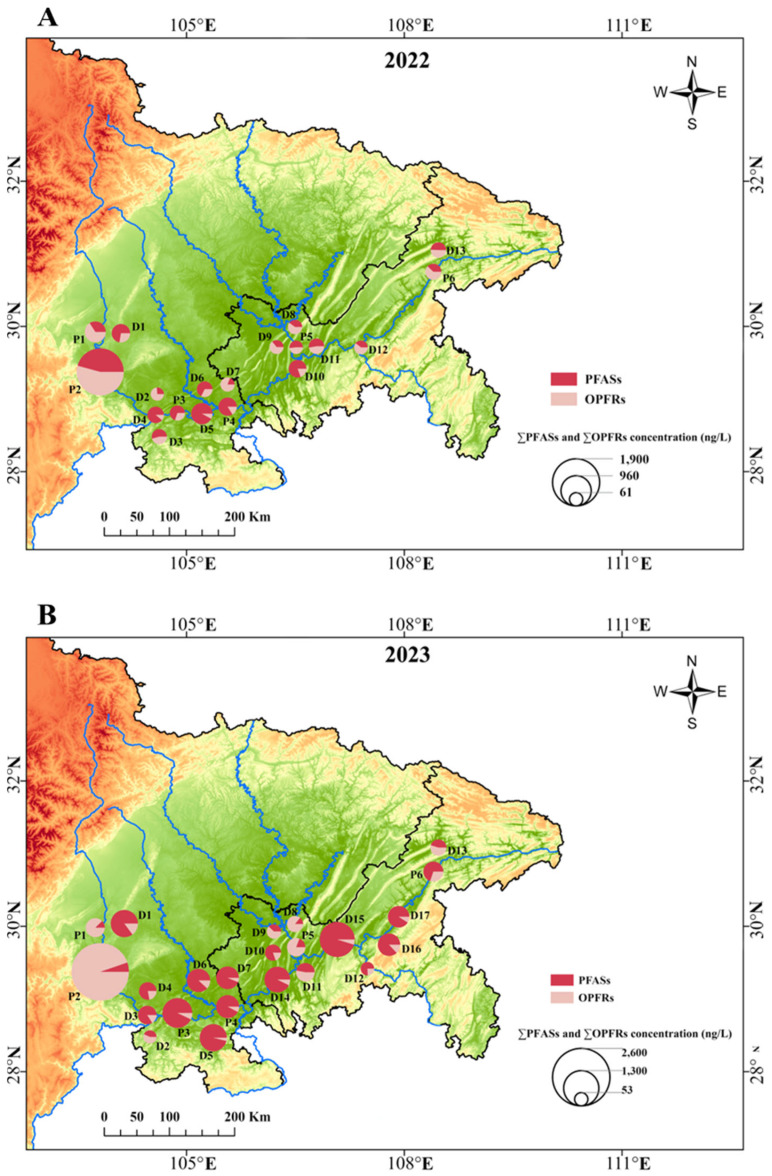
Cumulative concentrations of PFASs and OPFRs in the samples collected during 2022 (**A**) and 2023 (**B**) in the upper Yangtze River.

Figure 4 illustrates the changes in PFASs and OPFRs over time, highlighting significant differences in the cumulative concentration between the year of 2022 and 2023. Notably, the concentration of PFASs in 2023 was significantly higher than in 2022. NMDS analyses further confirmed the distinct differences in PFAS and OPFR concentrations between the two years (Figure S1). It is worth noting that before the sampling period in August 2023, heavy rainfall and flooding occurred in some areas of Sichuan and Chongqing, which may have affected the concentration of pollutants in the surface water. A previous study showed that higher concentration levels of PFASs in rivers during the rainy season could be attributed to the abundant precipitation and runoff in urban rivers [68].

**Figure 5:**
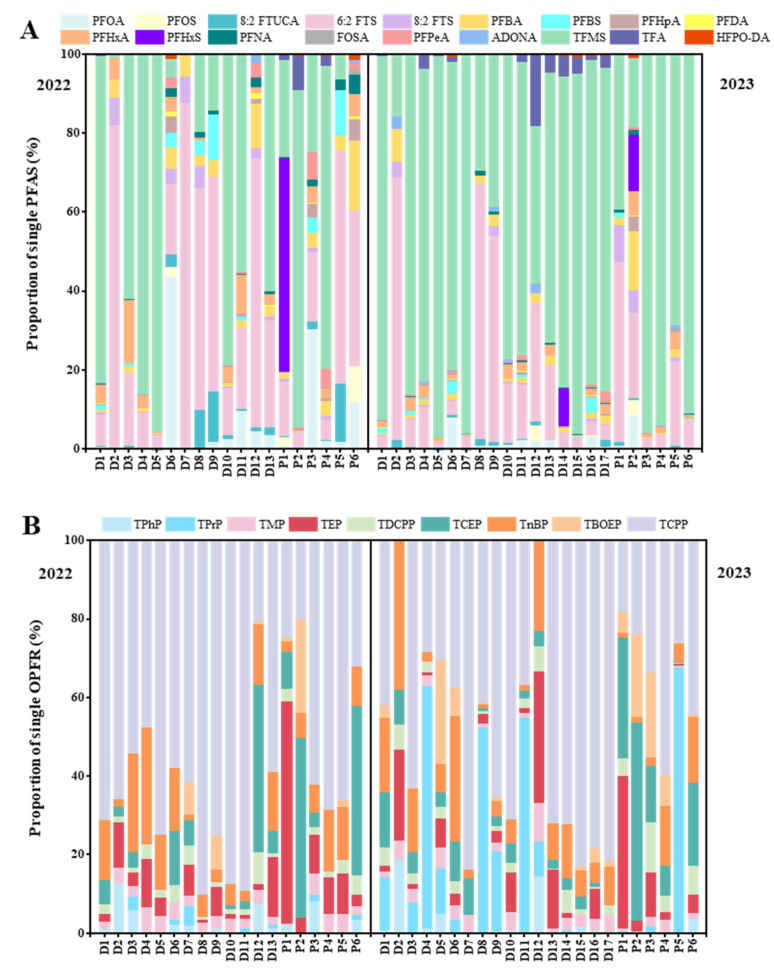
Relative contributions of individual composition to the total PFASs (**A**) and OPFRs (**B**) in water samples from the upper Yangtze River.

Figure 5 displays the composition profiles of individual PFASs and OPFRs. Among the PFASs, TFMS had with the largest contribution, ranging from 0–96% in 2022 and 16–97% in 2023. Furthermore, at some sampling points (D6, D7 in Luzhou, D8, D9 in Chongqing), the composition of PFASs also differed in 2022 and 2023 (Figure 5A). The PFASs detected in the waters of the upper Yangtze River were predominantly ultra-short-chain PFASs (e.g., TFMS, TFA) and FTS compounds (e.g., 6:2 FTS). This composition differed from previous reports on Yangtze River [33,34,45]. Recent studies have suggested that ultra-short-chain PFASs are widely prevalent in aquatic environments globally and are often found at higher concentrations than long-chain compounds [48], which aligns with the findings of this study. Halogenated OPFRs were the OPFRs that contribute the most to the surface water in the upper reaches of Yangtze River. TCPP was the dominant compound, contributing 20–90% in 2022 and 0–84% in 2023. Notably, there were significant differences in the composition of alkyl OPFRs in surface water in 2022 and 2023, especially TPrP (0–5% in 2022, 0–62% in 2023) and TnBP (1–30% in 2022, 2–38% in 2023). This may be partly attributed to the photolysis of aryl OPFRs due to the relatively low air temperature and UV intensity during August 2023, when heavy rains were experienced. These results are consistent with prior studies on OPFRs in the aquatic environments of Yangtze River [36,37].

### 3.3. Possible Source Analysis

Spearman analysis was conducted to explore the correlation between the concentration of PFASs and OPFRs [33,36], with the results shown in Tables S12 and S13 and Figure S2. Direct sources of PFASs include industrial production, transportation, use and disposal, and indirect sources are the degradation of precursor compounds [69]. Significant positive correlations were observed among certain PFASs in Yangtze River water samples (*p* < 0.05), suggesting the presence of common contamination sources. As shown in Table S12, strong correlation was identified between long-chain PFCAs (PFOA, PFNA, PFDA) and short-chain PFCAs (PFBA, PFPeA, PFHpA) (r > 0.60; *p* < 0.001). Additionally, HFPO-DA was strongly correlated with PFOS, PFBA, and PFPeA (r > 0.60; *p* < 0.001), further supporting the idea of shared contamination sources. There are several industrial parks related to the fluorine industry along Yangtze River, such as the fluorochemical industrial parks along the upper stream near Luzhou and Chongqing [34], which may be an important source of the PFOS, PFBA, PFPeA, and long-chain and short-chain PFCAs. Additionally, due to backward sewage treatment technology, industrial wastewater containing PFASs could easily enter Yangtze River and its drainage system. It was reported that large-scale fluorine chemical industrial parks may cause large-scale surface water pollution [70]. However, no significant correlations were detected between other PFASs. PFOA, PFNA, and PFDA are key precursors and processing aids in fluoropolymer production [71,72], while PFBA, PFPeA, and PFHpA are primarily used in the manufacture of products such as carpets, gloves, leather, ski wax, and outdoor textiles [73]. These findings suggested that the industries involved in producing precursors, processing aids, leather and textile products could be potential sources of these PFCAs. Municipal wastewater often contains high concentrations of PFASs due to the daily use of products containing PFASs and inefficiencies in wastewater treatment [74]. Serious PFAS contamination has been found in the inlet and outlet water of WWTPs in several cities along Yangtze River [34]. Given that the megacity of Chongqing is located along Yangtze River, there is a significant risk of PFASs in urban sewage being discharged into the upper Yangtze River.

No significant correlations were found between OPFRs in water bodies, suggesting that these compounds OPFRs may originate from diverse sources of contamination. Halogenated alkyl OPFRs (TCEP, TCPP, and TDCPP) are commonly used as flame retardants in furniture, electronic products, and textiles [55]. Meanwhile, alkyl OPFRs (TMP, TEP, TPrP, TnBP, and TBOEP) and aryl OPFRs (TPhP) are widely utilized as plasticizers, lubricants, and brighteners in textiles, building materials, and electronic products [75]. Therefore, the sources of OPFRs in the surface water of the upper Yangtze River are mainly caused by anthropogenic activities, including the use of building materials in daily life, the release of chemicals from household products, and industrial wastewater discharge. Additionally, atmospheric transport and sedimentation were the important sources of OPFRs (such as TCEP, TCPP, TBOEP, and TnBP) in surface water [76,77].

### 3.4. Estimated Annual Mass Loadings of PFASs and OPFRs in the Upper Yangtze River

Figure S3 and Table S14 show the estimated annual inflows of PFASs and OPFRs for the five major tributaries and the main stream. The flux (ton/year) of PFASs in the Yangtze River main stream was 50.93 in 2022 and 139.16 in 2023; in the Jinsha River, it was 1.73 in 2022 and 2.79 in 2023; in the Min River, it was 9.88 in 2022 and 34.08 in 2023; in the Tuo River, it was 1.27 in 2022 and 5.70 in 2023; in the Jialing River, it was 1.97 in 2022 and 2.10 in 2023; in the Wu River, it was 1.20 in 2022 and 1.64 in 2023. Therefore, the tributary that contributed the most to PFASs in the mainstream is the Minjiang River (Figure S3). The calculated annual emission loadings of TFMS, 6:2 FTS, PFOA, PFOS, PFBS, PFHxA and the total PFASs in the upper Yangtze River in 2022 were 41.72 tons, 5.84 tons, 0.89 tons, 0.0 tons, 0.04, 1.31 tons, and 50.93 tons, respectively. And these values of TFMS, 6:2 FTS, PFOA, PFOS, PFBS, PFHxA and the total PFASs in 2023 were 121.04 tons, 6.31 tons, 0.93 tons, 0.09 tons, 0.67 tons, 1.47 tons, and 139.16 tons, respectively. Except for TFMS, these values were lower than previously estimated annual emission fluxes from Yangtze River to the East China Sea (PFOA: 6.8 tons; PFOS: 0.88 tons; PFHxA: 2.2 tons; PFBS: 8.2 tons, PFASs: 20.7 tons) [33], suggesting that the middle and lower Yangtze River contributed the majority of PFASs. Although the PFAS concentration in the upper Yangtze River (max. values for PFOA and PFOS were 57.22 and 3.24 ng/L) were much lower than those in the majority rivers in the world (in Japan, PFOA and PFOS were 107 ng/L and 143 ng/L; in Florida, USA, PFOA and PFOS were 81 and 1135 ng/L) [78,79], the mass loading of PFASs in the upper Yangtze River were still higher than those in other rivers due to its huge annual water flow (annual mass loading of PFOA and PFOA in Japan were 0.045 tons and 0.060 tons) [79]. Considering the persistence and bioaccumulation, PFASs in the upper Yangtze River would be transported to the middle and lower reaches, and the East China Sea, and eventually enter the ocean, which could have significant environmental impacts.

For OPFRs, the flux (ton/year) in the main stream was 17.85 in 2022 and 20.49 in 2023; in the Jinsha River, it was 4.84 in 2022 and 3.64 in 2023; in the Min River, it was 5.00 in 2022 and 6.12 in 2023; in the Tuo River, it was 0.61 in 2022 and 0.77 in 2023; in the Jialing River, it was 3.19 in 2022 and 7.23 in 2023; in the Wu River, it was 1.84 in 2022 and 0.53 in 2023. It is obvious that the Minjiang River and Jialing River contribute significantly to the annual mass loadings of mainstream OPFRs (Figure S3). The calculated annual emission loadings of total OPFRs in the upper Yangtze River were lower than that from Yangtze River to the East China Sea (97.0 tons) [36]. Therefore, the middle and lower reaches may be the main contributors to OPFRs in Yangtze River, which may be related to the high population density and developed industry in the middle and lower reaches [80].

### 3.5. Ecological Risks Assessment

**Figure 6:**
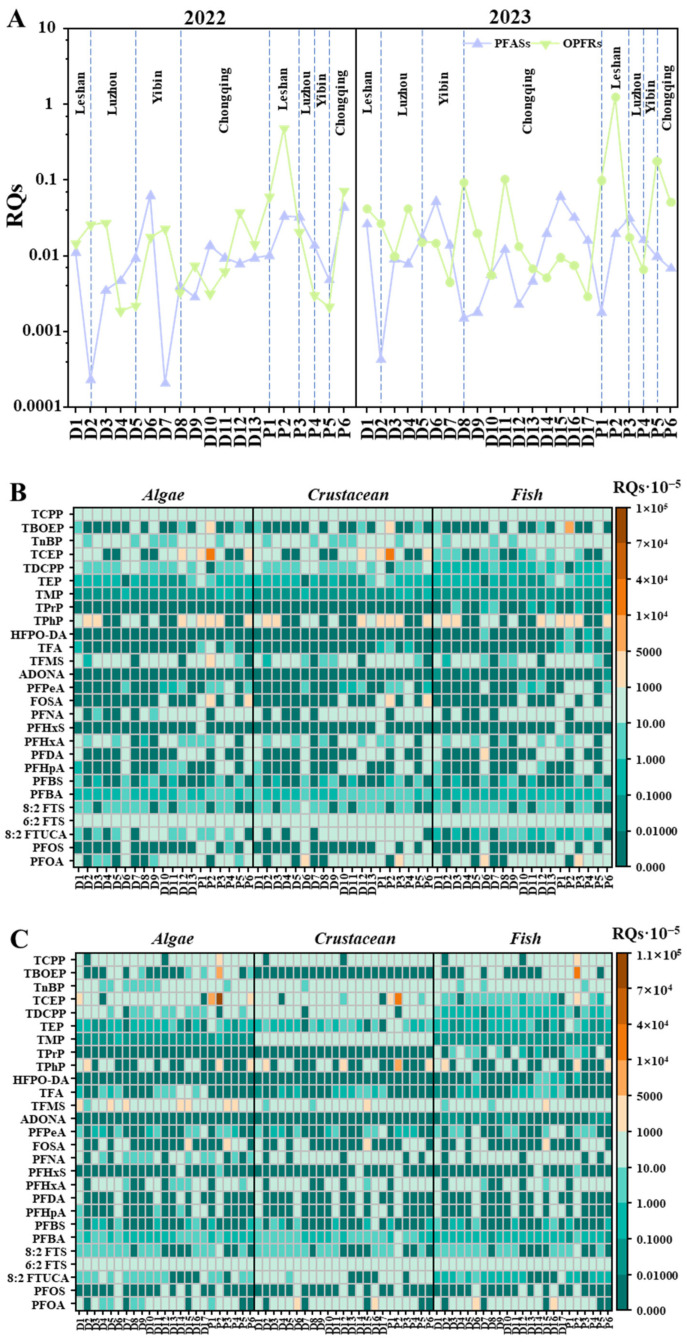
Risk quotients of PFASs and OPFRs. (**A**) The total risk quotients (RQ_STU_). Ecological risks related to individual compounds in 2022 (**B**) and 2023 (**C**) for algae, crustaceans, and fish in each water sample.

The ecological risks of individual and total RQs of PFASs and OPFRs in water samples were evaluated based on previously reported PNECs [81,82,83,84,85,86,87,88,89,90,91,92,93,94], with the results presented in Table S15 and Figure 6. Overall, the RQ_STU_ (summed risk quotient) values for both PFASs and OPFRs across the stream sampling points in all cities ranged between 0.01 and 0.1 (Figure 6A), indicating a low ecological risk in the upper Yangtze River. However, the RQ_STU_ of OPFRs at some sampling points in Chongqing suggested a medium risk (RQ: 0.1–1). Notably, a high risk (RQ > 1) of OPFRs was detected near the wastewater effluent in Leshan. Given the potential adverse effects of PFASs and OPFRs in surface waters, further studies on the chronic toxicity of these pollutants in aquatic organisms are essential for more comprehensive risk assessments, particularly for OPFRs.

As shown in Figure 6B,C, all PFASs were unlikely to have serious adverse effects on aquatic organisms (RQs < 0.1). Although PFASs in the upper Yangtze River appear to be relatively risk-free at present, their presence should not be ignored because of the persistence, accumulation, and migration. Moreover, for the main PFASs detected, TFMS and 6:2FTS, due to the lack of toxicity studies on ultra-short-chain PFASs and FTS-related compounds, attention should be paid to their possible environmental impacts. For OPFRs, TPhP, TCEP, and TBOEP showed a low risk (0.01 < RQs < 0.1) to three typical organisms at some points in 2022 (Figure 6B). However, TCEP presented a medium risk (0.1 < RQs < 1) for Algae at some sampling points (Figure 6C). The relatively higher ecological risks of TPhP, TCEP, and TBOEP were partly due to their high acute toxicity to aquatic organisms and relatively low PNEC values (Table S6). Compared with previous studies, the ecological risk of OPFRs in the upper Yangtze River surface water was lower in this study [36,37]. Considering the long-term exposure, cumulative adverse effects, and bioaccumulation of OPFRs in aquatic environments [17,21], more studies on the chronic toxicity of OPFRs in aquatic organisms are needed for risk assessment, especially for TPhP, TCEP, and TBOEP.

### 3.6. Health Risk Assessment

**Figure 7:**
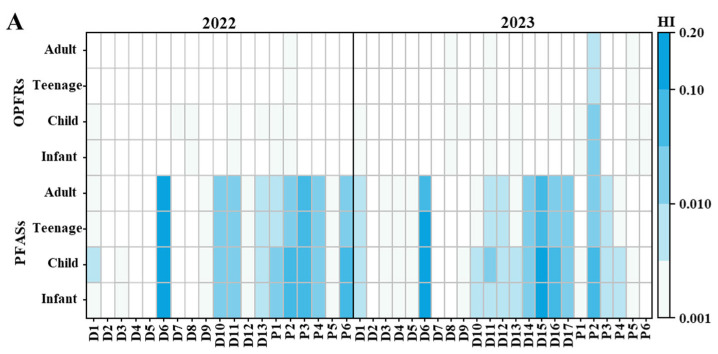

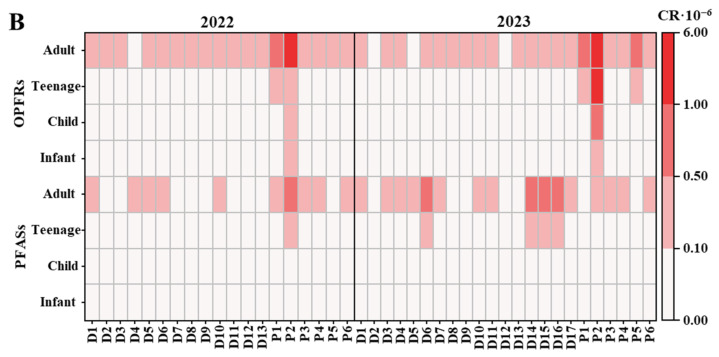
Health risk assessment. (**A**) Non-carcinogenicity (HI) and (**B**) carcinogenicity risk (CR) in each water sample.

Health risks from chemicals in aquatic environments are often linked to the human ingestion of drinking water [17,95]. Since some of the sampling points in this study were near water sources, drinking water intake was chosen as the basis for the health risk assessment. The results of hazard indices (His) and carcinogenic risks (CRs) for health-related PFASs and OPFRs are presented in Figure 7 and Table S16. For both individual compounds and the total concentration of PFASs (∑PFASs) and OPFRs (∑OPFRs), the non-carcinogenic (HI < 0.2) risk values were below theoretical risk (HI < 1) thresholds, suggesting that exposure to PFASs and OPFRs via surface water is likely safe for local residents. In general, the non-carcinogenic (non-CR) risk of PFASs was higher than that of OPFRs (Figure 7A). Notably, the non-carcinogenic risk of PFASs in Luzhou (Tuo River) and Chongqing (mainstream) was higher than that at other sampling points (Figure 7A). Regarding the age groups, the non-carcinogenic risk for PFASs followed the order: adult (2.50 × 10^−1^ in 2022 and 4.91 × 10^−1^ in 2023) > child (1.76 × 10^−1^ in 2022 and 1.60 × 10^−1^ in 2023) > teenage (1.13 × 10^−1^ in 2022 and 1.03 × 10^−1^ in 2023) > infant (6.70 × 10^−2^ in 2022 and 1.45 × 10^−1^ in 2023) (Table S16). The non-carcinogenic risk for OPFRs followed: child (1.48 × 10^−3^ in 2022 and 2.97 × 10^−3^ in 2023) > infant (1.34 × 10^−3^ in 2022 and 2.69 × 10^−3^ in 2023) > teenage (9.50 × 10^−4^ in 2022 and 1.90 × 10^−3^ in 2023) > adult (9.67 × 10^−4^ in 2022 and 1.43 × 10^−3^ in 2023) (Table S16). The non-CR risk level of PFASs in this study was higher than that of Chongqing surface water [37], while the non-CR risk level of OPFRs was lower than that of previous studies on Yangtze River surface water [32].

For carcinogenic risk, the CR values of PFASs at all sampling points were significantly lower than 10^−6^, indicating that the CR values associated with these pollutants within are acceptable levels. However, OPFRs at the Leshan (Min River) sampling points were estimated to pose a potential cancer risk to adults (10^−6^ to 10^−4^) (Figure 7B). The CR order for different age groups for PFASs was as follows: adult (9.96 × 10^−8^ in 2022 and 1.34 × 10^−7^ in 2023) > teenage (9.41 × 10^−8^ in 2022 and 1.26 × 10^−7^ in 2023) > child (5.18 × 10^−8^ in 2022 and 6.95 × 10^−8^ in 2023) > infant (2.58 × 10^−8^ in 2022 and 3.91 × 10^−8^ in 2023) (Table S16). The carcinogenic risk for OPFRs was as follows: adult (5.28 × 10^−7^ in 2022 and 1.66 × 10^−7^ in 2023) > teenage (1.10 × 10^−7^ in 2022 and 1.57 × 10^−7^ in 2023) > child (5.72 × 10^−8^ in 2022 and 8.66 × 10^−8^ in 2023) > infant (2.53 × 10^−8^ in 2022 and 3.14 × 10^−8^ in 2023) (Table S16). The health risks of OPFRs in these results were consistent with those in the middle and lower reaches of Yangtze River [30,31]. In order to fully elucidate the future exposure risks of PFASs and OPFRs to humans, more target compounds and their spatiotemporal variation characteristics need to be identified and studied. In addition, the available parameters (e.g., RfD and SF) of many PFASs and OPFRs are still unknown, which will lead to an underestimation of the associated risks.

## 4. Conclusions

Generally, the total concentration of PFASs in the upper Yangtze River ranged from 16.07 to 1231.11 ng/L, and OPFRs ranged from 17.36 to 190.42 ng/L. Ultra-short-chain PFASs (TFMS: ND to 1119.56 ng/L) were the most prevalent PFASs in the waters of the upper reaches of Yangtze River, while 6:2FTS and PFBA were prevalent in all samples. Halogenated alkyl OPFRs (TCPP and TCEP) were commonly found in the samples, especially in the effluent wastewater, with the highest concentrations reaching 1221.10 and 572.81 ng/L. Human activities and WWTP wastewater may be the main sources of PFASs and OPFRs in the samples. Furthermore, seasonal and hydrological factors further influenced the distribution patterns, with higher pollutant loads observed during the rainy season, likely due to increased runoff and urban wastewater overflow. In the upper Yangtze River, the concentration of PFASs in the main stream was higher than that in the tributaries, while there was no significant difference in OPFRs between the main stream and tributaries. The annual mass load calculations demonstrated that Minjiang River and Jialing River contribute significantly to PFASs and OPFRs in the main stream. The ecological risk assessments indicated that PFASs and OPFRs at most sampling sites posed low-to-moderate risks to aquatic organisms, including algae, crustaceans, and fish. However, medium ecological risks were observed for specific OPFRs, such as TCPP and TCEP, particularly near wastewater discharge points in regions with intensive industrial activities. Human health risk assessments revealed that non-carcinogenic risks from PFASs and OPFRs were generally below acceptable thresholds for all age groups. However, the carcinogenic risk of the adults associated with OPFRs was elevated at certain locations, particularly in Leshan City along the Min River. Therefore, it is crucial to enhance the supervision of PFASs and OPFRs in surface water while emphasizing the need for improved wastewater treatment technologies to mitigate the environmental and health impacts of these pollutants. Future research should focus on understanding the chronic toxicity and bioaccumulation of ultra-short-chain PFASs and halogenated alkyl OPFRs to support accurate ecological risk assessments and management policies.

## Figures and Tables

**Table 1:** Summary of PFAS and OPFR concentration (ng/L) in water samples.

Period	Compound	Surface Water					Effluent Wastewater				
		Min. (ng/L)	Max. (ng/L)	Median (ng/L)	Mean (ng/L)	DF (%)	Min. (ng/L)	Max. (ng/L)	Median (ng/L)	Mean (ng/L)	DF (%)
2022	Per- and poly-fluoroalkyl substances (PFASs)										
	PFOA	ND	57.22	1.93	6.25	69.2	ND	34.30	6.42	8.75	83.3
	PFOS	ND	3.24	3.24	0.25	7.7	ND	7.57	3.60	2.52	66.7
	8:2 FTUCA	ND	4.95	1.07	1.48	76.9	ND	5.39	0.62	1.49	83.3
	6:2 FTS	13.22	28.45	19.44	20.68	100.0	14.13	34.31	21.95	23.97	100.0
	8:2 FTS	ND	5.38	0.88	1.20	76.9	ND	2.96	1.48	0.89	50.0
	PFBA	0.72	6.92	1.71	2.18	100.0	1.35	14.36	4.10	6.04	100.0
	PFBS	ND	4.95	2.69	1.22	46.2	ND	4.39	4.16	1.46	50.0
	PFHpA	ND	5.30	0.63	0.56	38.5	ND	4.25	3.82	1.73	50.0
	PFDA	ND	1.53	0.45	0.21	30.8	ND	0.65	0.29	0.20	50.0
	PFHxA	ND	14.66	5.09	4.71	84.6	ND	6.82	4.65	2.87	83.3
	PFHxS	ND	ND	ND	ND	0.0	ND	87.55	87.55	14.59	16.7
	PFNA	ND	2.86	0.70	0.58	69.2	ND	3.86	0.99	1.32	83.3
	FOSA	ND	0.12	0.12	0.01	7.7	ND	1.27	0.63	0.44	66.7
	PFPeA	ND	3.59	0.81	0.42	30.8	ND	13.03	5.62	4.23	66.7
	ADONA	ND	0.67	0.65	0.13	23.1	ND	0.57	0.57	0.10	16.7
	TFMS	ND	433.46	53.49	91.29	84.6	ND	726.98	39.58	168.31	83.3
	TFA	ND	0.60	0.60	0.05	7.7	ND	72.62	7.26	13.61	50.0
	HFPO-DA	ND	1.14	1.14	0.09	7.7	ND	3.03	1.11	0.77	50.0
	∑PFASs	16.07	454.68	90.27	131.43	100.0	36.41	849.82	136.97	253.27	100.0
	organophosphate flame retardants (OPFRs)										
	TPhP	ND	5.84	1.78	1.66	61.5	ND	6.96	4.47	3.27	66.7
	TPrP	ND	4.19	1.18	0.76	38.5	ND	1.52	1.14	0.38	33.3
	TMP	1.73	2.94	2.01	2.03	100.0	1.72	3.01	2.46	2.36	100.0
	TEP	ND	14.04	2.31	3.34	92.3	3.50	176.51	4.94	38.01	100.0
	TDCPP	0.50	4.33	0.88	1.58	100.0	ND	10.43	1.18	3.32	83.3
	TCEP	ND	22.14	5.22	4.15	69.2	ND	461.48	42.80	91.48	66.7
	TnBP	0.87	19.03	5.51	7.09	100.0	3.36	64.86	8.31	17.12	100.0
	TBOEP	ND	7.46	2.85	1.03	30.8	ND	240.24	3.42	40.70	50.0
	TCPP	10.65	75.57	46.27	43.98	100.0	22.38	200.86	39.59	68.18	100.0
	∑OPFRs	26.25	93.19	62.90	65.62	100.0	33.95	1007.79	92.96	264.83	100.0
2023	Per- and poly-fluoroalkyl substances (PFASs)										
	PFOA	ND	43.19	1.61	4.90	82.4	ND	11.80	1.55	2.94	66.7
	PFOS	ND	2.20	ND	0.45	23.5	ND	5.26	ND	0.88	16.7
	8:2 FTUCA	ND	8.14	0.49	1.01	76.5	ND	0.78	0.58	0.52	83.3
	6: 2 FTS	15.15	38.36	20.11	21.37	100.0	14.17	29.11	21.74	21.58	100.0
	8:2 FTS	ND	4.57	0.84	0.89	70.6	ND	7.76	0.41	2.16	50.0
	PFBA	0.66	10.43	2.34	3.72	100.0	0.94	20.52	1.62	4.66	100.0
	PFBS	ND	20.56	ND	3.20	41.2	ND	0.70	ND	0.23	33.3
	PFHpA	ND	2.29	ND	0.56	35.3	ND	4.81	ND	0.80	16.7
	PFDA	ND	0.92	ND	0.16	29.4	ND	0.29	ND	0.05	16.7
	PFHxA	ND	13.37	3.72	4.69	76.5	ND	8.48	6.00	5.29	83.3
	PFHxS	ND	70.66	ND	4.16	5.9	ND	19.41	ND	3.24	16.7
	PFNA	ND	1.27	0.31	0.42	82.4	ND	1.82	0.19	0.41	66.7
	FOSA	ND	2.61	ND	0.20	29.4	ND	0.71	0.06	0.24	50.0
	PFPeA	ND	13.82	0.35	1.74	52.9	ND	0.98	0.20	0.39	50.0
	ADONA	ND	1.34	ND	0.40	52.9	ND	1.01	0.23	0.32	50.0
	TFMS	3.59	1119.56	260.44	337.15	100.0	18.61	888.25	161.47	297.23	100.0
	TFA	ND	51.44	2.79	8.36	64.7	ND	ND	ND	ND	0.0
	HFPO-DA	ND	7.56	ND	0.74	29.4	ND	0.92	ND	0.15	16.7
	∑PFASs	23.13	1231.11	302.65	394.12	100.0	47.45	926.93	220.70	341.09	100.0
	organophosphate flame retardants (OPFRs)										
	TPhP	ND	5.71	1.18	0.89	52.9	ND	13.57	9.44	3.15	33.3
	TPrP	ND	105.04	9.76	17.02	58.8	ND	189.98	2.74	32.80	66.7
	TMP	1.49	2.64	1.83	1.93	100.0	1.70	2.77	1.82	1.96	100.0
	TEP	ND	17.74	2.34	3.45	82.4	0.88	129.65	7.16	35.14	100.0
	TDCPP	ND	5.62	1.16	1.73	94.1	ND	15.53	8.96	6.21	66.7
	TCEP	ND	17.77	2.10	3.12	94.1	0.56	1221.10	19.04	227.55	100.0
	TnBP	0.83	23.93	3.96	7.01	100.0	1.59	34.42	10.26	13.90	100.0
	TBOEP	ND	8.76	1.90	1.41	47.1	ND	514.91	15.04	91.86	83.3
	TCPP	ND	86.19	40.32	39.81	88.2	23.36	572.81	59.78	136.06	100.0
	∑OPFRs	17.36	190.42	61.64	76.35	100.0	46.83	2427.21	206.76	548.61	100.0

DF: detection frequency. ND: not detected. Median: the middle concentration measurement such that half of the measurements are above it and half are below. Mean: represents the sum of all concentration measurements divided by the number of measurements (sampling points in this study). Detection frequency: refers to the proportion of measurements where the concentration exceeds the analytical method’s detection limit.

## Data Availability

Data are available in the article.

## Supplementary Materials

The following supporting information can be downloaded at: https://www.mdpi.com/article/10.3390/toxics13020116/s1, Text S1. Chemicals; Text S2. Sample preparation; Table S1. Summary of physico-chemical information of PAFAs and OPFRs in this study; Table S2. Sampling point information and water quality parameters; Table S3. The instrument details and gradient elution conditions; Table S4. LODs and LOQs of PFASs and OPFRs; Table S5. The runoff (108 m^3^) of the upper Yangtze River and its tributaries; Table S6. Toxicity values and risk quotients (RQs) for the aquatic organisms; Table S7. Summary of the available RfD (ng/kg/day) and SF (1/(ng/kg/day)); Table S8. Parameters used in human exposure estimation and health risk assessments; Table S9. Summary of the median and range (ng/L) of PFASs in water reported in the published literature; Table S10. Summary of the median and range (ng/L) of OPFRs in water reported in the published literature; Table S11. The chemical composition (organic contaminants) of the main industrial effluent wastewater; Table S12. Spearman rank order correlation coefficients of PFASs in water samples; Table S13. Spearman rank order correlation coefficients of OPFRs in water samples; Table S14. Annual emission flux (F: tons/year) of PFASs and OPFRs discharged into the upper Yangtze River; Table S15. Risk quotients (RQs) of PFASs and OPFRs for the aquatic organisms in the upper Yangtze River; Table S16. Non-carcinogenic risk (HI values) and carcinogenic risk (CR) of PFASs and OPFRs in the upper Yangtze River; Figure S1. NMDS analyses of PFAS (A) and OPFR (B) concentrations in water samples in 2022 and 2023; Figure S2. The calculated correlation between each concentration of PFASs (A) and OPFRs (B) based on non-parametric measure of the Spearman correlation; Figure S3. Annual emission flux (F: tons/year) of PFASs and OPFRs.
