# Supplementary material for: Per- and Poly-Fluoroalkyl Substances, and Organophosphate Flame Retardants in the Upper Yangtze River: Occurrence, Spatiotemporal Distribution, and Risk Assessment
Source: Toxics. 2025 Feb 1;13(2):116. doi: 10.3390/toxics13020116 (PMC11860806; doi:10.3390/toxics13020116)
Supplement: Supplementary file 1 [file toxics-13-00116-s001.zip › toxics-3411546-supplementary.pdf]

Supplementary Materials for

Per- and Poly-Fluoroalkyl Substances, and Organophosphate Flame Retardants in the Upper Yangtze River: Occurrence, Spatiotemporal Distribution, and Risk Assessment

Wen Sun [1,2], Zhiyou Fu [2], Yueyue Liu [2], Yingchen Bai [2], Yuyan Zhao [1], Chen Wang [2,*] and Fengchang Wu [2,*]

[1] College of Geo-Exploration Science and Technology, Jilin University, Changchun, China
[2] State Key Laboratory of Environmental Criteria and Risk Assessment, Chinese Research Academy of Environmental Sciences, Beijing 100012, China.
* Correspondence: wangchen@craes.org.cn (C.W.); wufengchang@vip.skleg.cn (F.W)

Text S1. Chemicals

The 18 target PFASs in this study can be divided into: long-chain perfluoroalkyl carboxylic acids (long-chain PFCAs: C8-C10), long-chain perfluoroalkyl sulfonic acids (long-chain PFSAs: C6-C8), short-chain PFCAs (C4-C7), short-chain PFSAs (C4), ultra-short-chain PFCAs (C2), ultra-short-chain PFSAs (C1), fluorotelomer sulfonic acids (FTSs), and other PFASs. Long-chain PFCAs: PFOA, PFNA, PFDA; long-chain PFSAs: PFHxS, PFOS; short-chain PFCAs: PFBA, PFPeA , PFHxA, PFHpA; short-chain PFSAs: PFBS; ultra-short-chain PFCAs: TFA; ultra-short-chain PFSAs: TFMS; FTSs: 6:2 FTS, 8:2 FTS; other PFASs: 8:2 FTUCA, FOSA, ADONA, HFPO-DA.

The nine target OPFRs in this study can be divided as follows: alkyl OPFRs (TMP, TEP, TPrP, TnBP, and TBOEP), halogenated alkyl OPFRs (TCEP, TCPP, and TDCPP), and aryl OPFRs (TPhP).

Text S2. Sample preparation

PFASs: After filtration, each 500 mL water sample was spiked with 50 ng of an internal standard mixture (MPFOA, MPFOS, and MPFHxA) by adding 500 μL of a 100 μg/L solution. Each WAX extraction cartridge was preconditioned sequentially with 4 mL of 0.1% NH_4OH in methanol, 4 mL of methanol, and 4 mL of Milli-Q water. The water samples were then loaded onto the cartridges at a rate of approximately one drop per second. Following sample loading, the cartridges were dried under vacuum, and elution was carried out sequentially with 4 mL of methanol and 4 mL of 0.1% NH_4OH in methanol. The resulting extracts were concentrated to dryness under a nitrogen stream, reconstituted in 500 μL of methanol, and filtered through a 0.22 μm nylon filter into 1 mL amber sample vials. The vials were then stored at -20°C until analysis.

OPFRs: After filtration, each 500 mL water sample was spiked with 50 ng of an internal standard mixture (d_{15}-TPHP, d_{27}-TBP, and d_{15}-TDCPP) by adding 500 μL of a 100 μg/L solution. Each HLB extraction cartridge was preconditioned sequentially with 6 mL of methanol, and 6 mL of Milli-Q water. The water samples were then loaded onto the cartridges at a rate of approximately one drop per second. Following sample loading, the cartridges were dried under vacuum, and elution was carried out sequentiall y with 10 mL of methanol. The resulting extracts were concentrated to dryness under a nitrogen stream, reconstituted in 500 μL of methanol, and filtered through a 0.22 μm nylon filter into 1 mL amber sample vials. The vials were then stored at -20°C until analysis.

Table S1. Summary of physico-chemical information of PAFAs and OPFRs in this study.

Compound name	Acronym	CAS number	Formula	Mass	pKa	Log Kow
Per- and poly-fluoroalkyl substances	***PFASs***					
Perfluorooctanoic acid	PFOA	335-67-1	$C_8HF_{15}O_2$	414.1	3.15	3.1
Perfluorooctane sulfonat	PFOS	1763-23-1	$C_8HF_{17}O_3S$	500.1	-1.64	5.61
2H-Perfluoro-2-decenoic Acid	8:2 FTUCA	70887-84-2	$C_{10}H_2F_{16}O_2$	458.1	8.72	5.93
6:2 fluorotelomer sulfonic acid	6:2 FTS	27619-97-2	$C_8H_5F_{13}O_3S$	428.1	1.23	3.73
8:2 fluorotelomer sulfonic acid	8:2 FTS	39108-34-4	$C_{10}H_5F_{17}O_3S$	538.2	-0.55	5.22
Perfluorobutanoic acid	PFBA	375-22-4	$C_4F_7O_2H$	214.2	-0.21	1.43
Perfluorobutanesulfonic acid	PFBS	375-73-5	$C_4HF_9O_3S$	300.1	-1.61	2.79
Perfluoroheptanoic acid	PFHpA	375-85-9	$C_7F_{13}O_2H$	364.1	0.06	2.05
Perfluorodecanoic acid	PFDA	335-76-2	$C_{10}F_{19}O_2H$	514.1	0.40	4.15
Perfluorohexanoic acid	PFHxA	307-24-4	$C_6F_{11}O_2H$	314.2	0.20	2.85
Perfluorohexanesulfonate	PFHxS	108427-53-8	$C_6F_{13}O_3S$	399.1	-1.64	3.48
Perfluorononanoic acid	PFNA	375-95-1	$C_9F_{17}O_2H$	464.1	0.23	3.54
Perfluorooctane sulfonamide	FOSA	754-91-6	$C_8H_2F_{17}O_2NS$	499.1	-1.30	6.44
Perfluoropentanoic acid	PFPeA	2706-90-3	$C_5F_9O_2H$	264.2	-0.80	1.35
4,8-dioxa-3H-Perfluorononanoic acid	ADONA	919005-14-4	$C_7H_2F_{12}O_4$	378.1	-0.22	5.74
Trifluoromethanesulfonic acid	TFMS	1493-13-6	CHF_3O_3S	150.1	0.03	-0.2
Trifluoroacetic acid	TFA	76-05-1	$C_2HF_3O_2$	114.1	0.23	0.76
Hexafluoropropylene oxide dimer acid	HFPO-DA	13252-13-6	$C_6HF_{11}O_3$	330.1	-0.73	5.41
Internal standards for PFASs						
Perfluoro-n-[1,2,3,4-$^{13}C_4$] octanoic acid	MPFOA	960315-48-4	$^{13}C_4{}^{12}C_4HF_{15}O_2$	418.0	-	-
Sodium Perfluoro-1-[1,2,3,4-$^{13}C_4$] octanesulfonate	MPFOS	960315-53-1	$^{13}C_4{}^{12}C_4F_{17}NaO_3S$	526.1	-	-
Perfluoro [1,2-$^{13}C_2$] hexanoate acid	MPFHxA	960315-47-3	$^{13}C_2{}^{12}C_4HF_{11}O_2$	316.0	-	-
organophosphate flame retardants	***OPFRs***					
Triphenyl Phosphate	TPhP	115-86-6	$C_{18}H_{15}O_4P$	326.3	1.27	4.59
Tripropyl Phosphate	TPrP	513-08-6	$C_9H_{21}O_4P$	208.2	1	1.78
Trimethyl Phosphate	TMP	512-56-1	$C_3H_9O_4P$	140.1	1.3	-0.19
Triethyl Phosphate	TEP	78-40-0	$C_6H_{15}O_4P$	182.2	1.29	0.8
Tris(2-chloro-1-(chloromethyl)ethyl)phosphate	TDCPP	13674-87-8	$C_9H_{15}Cl_6O_4P$	430.9	1.5	3.65
Tris(2-chloroethyl)phosphate	TCEP	115-96-8	$C_6H_{12}Cl_3O_4P$	285.5	1.5	1.44
Tributyl Phosphate	TnBP	126-73-8	$C_{12}H_{27}O_4P$	266.3	1.3	4
Tris(2-butyloxyethyl)phosphate	TBOEP	78-51-3	$C_{18}H_{39}O_7P$	398.5	1.3	3.65
Tris(2-chloroisopropyl) phosphate	TCPP	13674-84-5	$C_9H_{18}Cl_3O_4P$	327.6	-	2.33
Internal standards for OPFRs						
Triphenyl phosphate (D15)	d_{15}-TPHP	1173020-30-8	$C_{18}D_{15}O_4P$	314.4	-	-
Tributyl phosphate (D27)	d_{27}-TBP	61196-26-7	$C_{12}D_{27}O_4P$	293.5	-	-
Tris(1,3-dichloro-2-propyl) phosphate (D15)	d_{15}-TDCPP	1447569-77-8	$C_9D_{15}Cl_6O_4P$	446.0	-	-

Table S2. Sampling point information and water quality parameters.

City	Sampling point	River name	Longitude	Latitude	T (℃)	pH	ORP (mV)	DO (mg/L)	Conductivity (mS/cm)
**2022**									
Leshan(LS)	D1	Min	103.785°E	29.498°N	28.9	7.83	236.6	7.34	0.501
Yibin(YB)	D2	Jinsha	104.556°E	28.703°N	28.2	7.98	191.8	8.80	0.503
	D3	Min	104.627°E	28.779°N	28.7	8.02	198.9	7.04	0.495
	D4	Yangtze	104.728°E	28.783°N	25.2	8.08	207.9	7.75	0.491
Luzhou(LZ)	D5	Yangtze	105.358°E	29.621°N	29.4	7.58	236.7	6.51	0.494
	D6	Tuo	105.447°E	28.901°N	33.7	8.37	231.5	7.88	0.512
	D7	Yangtze	105.566°E	28.902°N	25.7	8.06	224.3	7.88	0.505
Chongqing(CQ)	D8	Jialing	106.518°E	29.763°N	33.7	7.89	195	7.13	0.501
	D9	Jialing	106.458°E	29.666°N	32.2	7.91	267.1	7.21	0.493
	D10	Yangtze	106.535°E	29.517°N	29.4	8.27	215.1	8.75	0.492
	D11	Yangtze	106.597°E	29.621°N	28.5	7.84	251.5	7.29	0.49
	D12	Wu	107.407°E	29.699°N	26.5	7.75	265	7.17	0.492
	D13	Yangtze	108.470°E	30.840°N	29.5	8.11	207.7	7.43	0.511
Leshan(LS)	P1	Min	103.746°E	29.533°N	27.2	7.18	235.7	6.54	0.484
	P2	Min	103.815°E	29.371°N	32.6	7.74	236.2	6.86	0.364
Yibin(YB)	P3	Yangtze	104.728°E	28.807°N	31.4	7.61	211.6	4.97	0.385
Luzhou(LZ)	P4	Yangtze	105.567°E	28.895°N	26.4	8.02	223.4	8.30	0.397
Chongqing(CQ)	P5	Jialing	106.511°E	29.715°N	32.2	8.78	230.9	7.47	0.404
	P6	Yangtze	108.401°E	30.753°N	29.1	8.04	165.8	6.74	0.404
**2023**									
Leshan(LS)	D1	Min	103.783°E	29.500°N	26.9	7.41	189.1	5.79	0.452
Yibin(YB)	D2	Jinsha	104.616°E	28.758°N	23.7	8.27	141.9	7.70	0.454
	D3	Min	104.625°E	28.780°N	24.2	7.74	167.3	7.26	0.465
	D4	Yangtze	104.728°E	28.782°N	23.2	7.92	131.1	7.22	0.472
Luzhou(LZ)	D5	Yangtze	105.369°E	29.780°N	26.7	7.86	122.4	7.14	0.469
	D6	Tuo	105.446°E	28.900°N	32.7	7.98	125.2	7.11	0.471
	D7	Yangtze	105.566°E	28.903°N	25.3	8.02	137.9	6.99	0.443
Chongqing(CQ)	D8	Jialing	106.496°E	29.779°N	28.4	7.73	108.0	6.84	0.452
	D9	Jialing	106.456°E	29.666°N	27.8	7.60	139.3	6.54	0.427
	D10	Yangtze	106.531°E	29.523°N	26.3	7.89	107.6	7.24	0.415
	D11	Yangtze	106.597°E	29.620°N	27.8	7.82	108.4	7.63	0.424
	D12	Wu	107.403°E	29.712°N	23.9	7.83	115.2	7.52	0.414
	D13	Yangtze	108.471°E	30.840°N	29.6	7.54	110.6	6.66	0.402
	D14	Yangtze	106.251°E	29.268	26.4	7.95	117.9	7.26	0.429
	D15	Yangtze	107.077°E	29.819	27.4	7.86	109.8	7.09	0.442
	D16	Yangtze	107.790°E	29.933	30.2	7.77	97.0	6.76	0.345
	D17	Yangtze	107.923°E	30.132	27.9	7.90	104.8	6.90	0.35
Leshan(LS)	P1	Min	103.744°E	29.535°N	26.6	7.23	240.7	5.70	0.296
	P2	Min	103.812°E	29.374°N	30.6	7.40	149.9	7.38	0.299
Yibin(YB)	P3	Yangtze	104.728°E	28.807°N	30.0	7.74	157.1	4.89	0.298
Luzhou(LZ)	P4	Yangtze	105.567°E	28.895°N	27.8	7.43	140.3	5.91	0.315
Chongqing(CQ)	P5	Jialing	106.511°E	29.713°N	28.3	7.70	144.1	6.49	0.322
	P6	Yangtze	108.401°E	30.753°N	29.6	7.70	73.1	6.78	0.356

Table S3. The instrument details and gradient elution conditions.

Category	Item	Condition
		LC
	Chromatographic column	Agilent Zorbax Eclipse Plus-C18（Rapid Resolution HD 2.1 × 150 mm，3.5 μm）
	Volume injected	5 μL
	Column temperature	30 ℃
	Flow rate	0.3 mL/min
	Mobile phase	A: Milli-Q water; (B) Methanol both with 2.5 mM Ammonium fluoride
PFASs	Mobile phase gradient	0 min (30 % B), 0.5 min (30 % B), 10 min (90 % B), 20 min (95 % B), and return to the initial conditions (equilibration time 12 min)
		MS-MS
	Ionisation	ESI (-)
	Gas Temperature	350℃
	Cone Gas Flow	10 mL/min
	Nebulizer pressure	35 psi
	Capillary voltage	3500 V
	delta EMV	400 V
	Measurement mode	MRM
		LC
	Chromatographic column	Agilent Zorbax Eclipse Plus-C18（Rapid Resolution HD 2.1 × 150 mm，3.5 μm）
	Volume injected	5 μL
	Column temperature	30 ℃
	Flow rate	0.3 mL/min
	Mobile phase	A: Milli-Q water; (B) Methanol both with 0.1% Formic Acid
	Mobile phase gradient	0 min (30% B), 0.5 min (30% B), 12 min (90% B), 18 min (98% B) and 25 min (98% B), and return to the initial conditions (equilibration time 15 min).
OPFRs		MS-MS
	Ionisation	ESI (+)
	Gas Temperature	300° C
	Cone Gas Flow	10 mL/min
	Nebulizer pressure	35 psi
	Capillary voltage	3500 V
	delta EMV	400 V
	Measurement mode	MRM

Table S4. LODs and LOQs of PFASs and OPFRs.

Compound	LOD(ng/L)	LOQ(ng/L)	Range(ng/mL)	R^2	Recovery rate
PFASs	0.020-0.94	0.068-1.43	1–200	≥0.99	67.8–113.4 %
OPFRs	0.029-0.14	0.044-0.45	1–500	≥0.99	79.3–105.2 %

Table S5. The runoff (10^8 m^3) of the upper Yangtze River and its tributaries.

Period	Jinsha River	Min River	Tuo River	Jialing River	Wu River	Yangtze River
2022	1074.10	591.12	97.30	471.44	353.99	2755.50
2023	1207.95	665.36	102.77	555.47	305.22	2800.90

Table S6. Toxicity values and risk quotients (RQs) for the aquatic organisms.

Compound	Organism	EC50/LC50 (mg/L)	PNEC (μg/L)	RQs (mean, 2022)	RQs (mean, 2023)	RQs (Max., 2022)	RQs (Max., 2023)
	Algae	7.58[1]	7.58[1]	8.25E-04	5.08E-04	7.55E-03	5.70E-03
PFOA	Crustacean	1.5[1]	1.5[1]	4.17E-03	2.56E-03	3.81E-02	2.88E-02
	Fish	1.34[1]	1.34[1]	4.67E-03	2.87E-03	4.27E-02	3.22E-02
	Algae	14.28[1]	14.28[1]	1.74E-05	1.77E-05	2.27E-04	1.54E-04
PFOS	Crustacean	3.13[1]	3.13[1]	7.96E-05	8.09E-05	1.03E-03	7.04E-04
	Fish	3.04[1]	3.04[1]	8.19E-05	8.33E-05	1.07E-03	7.25E-04
	Algae	7.58[2]	7.58[2]	1.96E-04	8.47E-05	6.53E-04	4.13E-04
8:2 FTUCA	Crustacean	32[2]	3.2[2]	4.64E-04	2.01E-04	1.55E-03	9.78E-04
	Fish	81[2]	81[2]	1.83E-05	7.93E-06	6.11E-05	3.86E-05
	Algae	125[3]	125[3]	1.65E-04	1.61E-04	2.28E-04	2.10E-04
6: 2 FTS	Crustacean	109[3]	109[3]	1.90E-04	1.84E-04	2.61E-04	2.41E-04
	Fish	107[3]	107[3]	1.93E-04	1.88E-04	2.66E-04	2.46E-04
	Algae	76.1[4]	76.1[4]	1.58E-05	7.87E-06	7.07E-05	1.75E-05
8:2 FTS	Crustacean	47.7[4]	47.7[4]	2.53E-05	1.26E-05	1.13E-04	2.79E-05
	Fish	69.8[4]	69.8[4]	1.73E-05	8.58E-06	7.71E-05	1.91E-05
	Algae	262.2[5]	262.2[5]	8.32E-06	9.02E-06	2.64E-05	3.66E-05
PFBA	Crustacean	183.64[5]	183.64[5]	1.19E-05	1.29E-05	3.77E-05	5.23E-05
	Fish	1200[6]	1200[6]	1.82E-06	1.97E-06	5.76E-06	8.01E-06
	Algae	351.89[1]	351.89[1]	3.47E-06	5.57E-06	1.41E-05	5.08E-05
PFBS	Crustacean	186.87[1]	186.87[1]	6.53E-06	1.05E-05	2.65E-05	9.56E-05
	Fish	344.67[1]	344.67[1]	3.54E-06	5.68E-06	1.44E-05	5.18E-05
	Algae	16.86[1]	16.86[1]	3.31E-05	1.54E-05	3.14E-04	1.36E-04
PFHpA	Crustacean	4.15[1]	4.15[1]	1.34E-04	6.24E-05	1.28E-03	5.51E-04
	Fish	4.35[1]	4.35[1]	1.28E-04	5.96E-05	1.22E-03	5.26E-04
	Algae	1.47[1]	1.47[1]	1.42E-04	6.12E-05	1.04E-03	5.66E-04
PFDA	Crustacean	0.19[1]	0.19[1]	1.10E-03	4.73E-04	8.03E-03	4.38E-03
	Fish	0.12[1]	0.12[1]	1.74E-03	7.49E-04	1.27E-02	6.94E-03
	Algae	36.83[7]	36.83[7]	1.28E-04	1.25E-04	3.98E-04	3.21E-04
PFHxA	Crustacean	11.31[7]	11.31[7]	4.16E-04	4.06E-04	1.30E-03	1.05E-03
	Fish	14[7]	14[7]	3.36E-04	3.28E-04	1.05E-03	8.44E-04
	Algae	220.4[4]	220.4[4]	-	-	-	-
PFHxS	Crustacean	190.4[4]	190.4[4]	-	-	-	-
	Fish	301.3[4]	301.3[4]	-	-	-	-
	Algae	3.35[1]	3.35[1]	1.73E-04	9.65E-05	8.53E-04	3.80E-04

PFNA	Crustacean	0.53[1]	0.53[1]	1.09E-03	6.10E-04	5.39E-03	2.40E-03
	Fish	0.41[1]	0.41[1]	1.41E-03	7.88E-04	6.97E-03	3.10E-03
	Algae	0.07[4]	0.07[4]	1.29E-04	1.79E-03	1.67E-03	1.01E-02
FOSA	Crustacean	0.08[4]	0.08[4]	1.13E-04	1.56E-03	1.46E-03	8.84E-03
	Fish	0.15[4]	0.15[4]	6.00E-05	8.33E-04	7.80E-04	4.71E-03
	Algae	81.7[2]	81.7[2]	5.18E-06	8.29E-06	4.40E-05	6.33E-05
PFPeA	Crustacean	112[2]	112[2]	3.78E-06	6.05E-06	3.21E-05	4.62E-05
	Fish	32[2]	32[2]	1.32E-05	2.12E-05	1.12E-04	1.62E-04
	Algae	-	-	-	-	-	-
ADONA	Crustacean	-	-	-	-	-	-
	Fish	2000[8]	2000[8]	6.65E-08	2.57E-07	3.34E-07	6.69E-07
	Algae	48[9]	48[9]	1.90E-03	6.11E-03	9.03E-03	1.85E-02
TFMS	Crustacean	100[10]	100[10]	9.13E-04	2.93E-03	4.33E-03	8.88E-03
	Fish	100[11]	100[11]	9.13E-04	2.93E-03	4.33E-03	8.88E-03
	Algae	120[12]	120[12]	3.86E-07	1.68E-05	5.02E-06	8.03E-05
TFA	Crustacean	1200[13]	1200[13]	3.86E-08	1.68E-06	5.02E-07	8.03E-06
	Fish	1200[13]	1200[13]	3.86E-08	1.68E-06	5.02E-07	8.03E-06
	Algae	-	-	-	-	-	-
HFPO-DA	Crustacean	-	-	-	-	-	-
	Fish	100[8]	100[8]	8.74E-07	1.39E-06	1.14E-05	2.37E-05
	Algae	0.30[14]	0.30[14]	5.53E-03	2.95E-03	1.95E-02	1.90E-02
TPhP	Crustacean	0.25[14]	0.25[14]	6.63E-03	3.54E-03	2.34E-02	2.28E-02
	Fish	0.32[14]	0.32[14]	5.18E-03	2.77E-03	1.82E-02	1.78E-02
	Algae	-	-	-	-	-	-
TPrP	Crustacean	-	-	-	-	-	-
	Fish	159.1[15]	159.1[15]	4.76E-06	1.07E-04	2.64E-05	6.60E-04
	Algae	2444[14]	2444[14]	8.31E-07	7.88E-07	1.20E-06	1.08E-06
TMP	Crustacean	3.6[14]	3.6[14]	5.64E-04	5.35E-04	8.18E-04	7.32E-04
	Fish	3625[14]	3625[14]	5.60E-07	5.31E-07	8.12E-07	7.27E-07
	Algae	900[14]	900[14]	3.71E-06	3.83E-06	1.56E-05	1.97E-05
TEP	Crustacean	350[14]	350[14]	9.54E-06	9.85E-06	4.01E-05	5.07E-05
	Fish	1250[14]	1250[14]	2.67E-06	2.76E-06	1.12E-05	1.42E-05
	Algae	12.0[14]	12.0[14]	1.32E-04	1.44E-04	3.61E-04	4.68E-04
TDCPP	Crustacean	3.8[14]	3.8[14]	4.17E-04	4.54E-04	1.14E-03	1.48E-03
	Fish	350[14]	350[14]	4.52E-06	4.93E-06	1.24E-05	1.61E-05
	Algae	1.2[14]	1.2[14]	3.46E-03	2.60E-03	1.84E-02	1.48E-02
TCEP	Crustacean	7.1[14]	7.1[14]	5.84E-04	4.39E-04	3.12E-03	2.50E-03
	Fish	90.0[14]	90.0[14]	4.61E-05	3.47E-05	2.46E-04	1.97E-04
	Algae	34.0[14]	34.0[14]	2.08E-04	2.06E-04	5.60E-04	7.04E-04
TnBP	Crustacean	11.0[14]	11.0[14]	6.44E-04	6.38E-04	1.73E-03	2.18E-03
	Fish	20.0[14]	20.0[14]	3.54E-04	3.51E-04	9.51E-04	1.20E-03
	Algae	9.49[14]	9.49[14]	1.09E-04	1.49E-04	7.86E-04	9.24E-04
TBOEP	Crustacean	-	-	-	-	-	-
	Fish	3.3[14]	3.3[14]	3.13E-04	4.28E-04	2.26E-03	2.66E-03
	Algae	47.0[14]	47.0[14]	9.36E-04	8.47E-04	1.61E-03	1.83E-03
TCPP	Crustacean	63.0[14]	63.0[14]	6.98E-04	6.32E-04	1.20E-03	1.37E-03
	Fish	30.0[14]	30.0[14]	1.47E-03	1.33E-03	2.52E-03	2.87E-03

Table S7. Summary of the available RfD (ng/kg/day) and SF (1/(ng/kg/day))

Compound	RfD (ng//kg/day)	Reference	SF (1/(ng/kg/day))	Reference
PFOA	20	IRIS	7.77E-08	CTV platform
PFOS	20	IRIS	9.28E-08	CTV platform
8:2 FTUCA	9580	CTV platform	9.64E-08	CTV platform
6:2 FTS	-	-	-	-
8:2 FTS	11200	CTV platform	9.57E-08	CTV platform
PFBA	22100	CTV platform	3.26E-07	CTV platform
PFBS	22100	CTV platform	3.26E-07	CTV platform
PFHpA	12300	CTV platform	8.68E-08	CTV platform
PFDA	10700	CTV platform	1.10E-07	CTV platform
PFHxA	12700	CTV platform	1.17E-07	CTV platform
PFHxS	9180	CTV platform	9.18E-08	CTV platform
PFNA	9830	CTV platform	8.84E-08	CTV platform
FOSA	20	IRIS	9.28E-08	CTV platform
PFPeA	14700	CTV platform	1.31E-07	CTV platform
ADONA	8750	CTV platform	1.07E-07	CTV platform
TFMS	111000	CTV platform	1.99E-08	CTV platform
TFA	19600	CTV platform	1.02E-07	CTV platform
HFPO-DA	3	[16]	-	-
TPhP	70000	[17]	4.09E-07	CTV platform
TPrP	4050	CTV platform	4.80E-08	CTV platform
TMP	10000	PPRTV	2.00E-08	PPRTV
TEP	130000	[17]	8.25E-08	CTV platform
TDCPP	20000	[18]	3.10E-08	[18]
TCEP	130000	[17]	8.25E-08	CTV platform
TnBP	10000	[17]	9.00E-09	[17]
TBOEP	15000	[17]	4.19E-08	CTV platform
TCPP	3090	CTV platform	1.80E-07	CTV platform

Table S8. Parameters used in human exposure estimation and health risk assessments.

Parameter	Unit	Value			
		Infant (< 2 y)	Child (2 ~ < 6)	Teenage (6 ~ < 18)	Adult (18~< 79)
IR: Ingestion rate	L/day	0.413	0.801	1.289	1.978
BW: Body weight	kg	8.867	15.548	39.045	63.4
EF: Exposure frequency	day/year	365	365	365	365
ED: Exposure duration	year	2	4	12	61
AT (non-CR): Averaging time for non-carcinogenic effect	day	730	1460	4380	22265
AT (CR): Averaging time for carcinogenic effect	day	28835	28835	28835	28835

All the risk assessment parameters were averaged and collected from the Exposure Factors Handbook of the Chinese Population and USEPA (1997).

Table S9. Summary of median and range (ng/L) of PFASs in water reported in the published literature.

Location	Yangtze River, China	Yellow River, China	Pearl River, China	Huai River, China	Taihu Lake, China	Poyang Lake, China	Thames River, Britain	Several rivers, Europe	Delaware River, USA
PFOA	12.2 (3.48-36.5)	2.45 (0.15-4.92)	1.82 (0.40-52.8)	6.01 (4.24-9.06)	22.04 (13.76-73.58)	6.5 (1.8-17)	8.46 (5.56-11.7)	2.71 (0.86-3.66)	5.24 (2.12-14.9)
PFOS	1.41 (0.36-12.12)	2.29 (0.09-4.40)	8.56 (1.38-23.57)	1.90 (0.48-3.72)	4.11 (1.30-12.91)	2.7 (1.4-21)	12.9 (8.12-18.8)	4.28 (0.23-8.56)	3.50 (0.97-6.92)
6:2 FTS	0.11 (ND-6.97)	0.04 (ND-0.24)	0.48 (0.09-1.28)	0.03 (ND-0.41)	2.76 (ND-94.25)	2.9 (0.91-7.6)	5.07 (2.25-13.9)	2.08 (0.04-11.3)	0.96 (0.04-2.79)
8:2 FTS	ND (ND-0.03)	ND	ND (ND-0.05)	-	-	0.02 (0.003-0.32)	0.09 (0.06-0.19)	0.04 (ND-0.18)	0.05 (ND-0.28)
PFBA	3.92 (0.93-9.61)	3.52 (1.20-7.38)	1.80 (0.88-9.40)	5.37 (3.91-22.8)	28.07 (11.15-76.27)	14 (7.2-530)	6.86 (4.62-9.79)	4.57 (0.84-6.17)	2.32 (1.47-6.51)
PFBS	2.22 (0.22-4.68)	0.45 (0.07-4.68)	2.47 (0.21-21.51)	0.74 (0.52-1.59)	16.39 (7.04-40.21)	2.2 (0.69-320)	5.27 (3.26-6.75)	20.1 (0.46-146)	1.92 (0.52-4.20)
PFHpA	0.76 (0.29-4.43)	0.56 (0.02-0.74)	0.42 (0.07-2.11)	0.87 (0.72-1.30)	3.92 (2.47-5.83)	0.92 (0.17-30)	4.15 (2.58-5.19)	1.22 (0.20-1.99)	2.02 (0.93-5.70)
PFDA	0.07 (0.03-1.59)	0.04 (ND-0.31)	0.18 (0.06-0.87)	0.23 (0.13-0.42)	0.76 (0.31-2.95)	0.17 (0.091-0.56)	0.85 (0.52-1.22)	0.31 (0.07-1.02)	0.83 (0.15-1.84)
PFHxA	1.07 (0.33-28.2)	1.01 (0.10-1.57)	0.58 (0.34-2.99)	1.20 (0.89-1.97)	23.45 (11.98-188.16)	0.72 (0.33-6.9)	12.7 (7.32-15.0)	2.95 (0.83-4.56)	7.78 (1.89-15.5)
PFHxS	3.11 (0.92-85.77)	7.88 (0.09-25.69)	0.62 (0.09-4.17)	0.15 (0.09-1.52)	4.82 (1.28-9.63)	0.20 (0.039-25)	6.42 (4.96-11.3)	2.03 (0.12-3.90)	1.72 (0.65-2.63)
PFNA	0.36 (0.15-2.75)	0.45 (0.05-0.76)	0.35 (0.22-1.28)	1.15 (0.77-1.35)	2.35 (1.44-4.61)	0.53 (0.31-0.88)	1.17 (0.77-1.71)	2.03 (0.12-3.91)	2.36 (0.76-4.81)
FOSA	ND (ND-0.62)	-	-	-	0.045 (0.019-0.13)	-	-	-	-

PFPeA	0.58 (0.21-3.60)	0.85 (0.04-1.13)	0.47 (0.15-3.68)	0.94 (0.58-1.25)	4.09 (2.61-6.35)	0.64 (ND-1.5)	16.1 (10.1-19.9)	2.63 (0.42-4.02)	6.14 (1.72-11.0)
ADONA	-	-	-	-	-	-	-	0.02 (ND-1.5)	-
TFMS	-	-	-	-	-	-	-	8.04 (1.28-2112.5)	5
TFA	-	-	-	-	-	-	-	1400 (400-6000)	293.7 (12.8-2194.6)
HFPO-DA	0.14 (ND-1.29)	1.30 (ND-1.74)	0.70 (0.21-10.3)	1.40 (0.83-3.62)	3.09 (0.54-9.79)	0.55 (0.29-5.7)	1.10 (0.70-1.58)	0.02 (ND-1.55)	2.02 (0.78-8.75)
Reference	[19,20]	[19]	[19]	[19]	[19,21]	[22]	[19]	[19,23]	[19,24]

Table S10. Summary of the median and range (ng/L) of OPFRs in water reported in the published literature.

Location	Yangtze River, China	Yellow River, China	Pearl River, China	Xiang River, China	Nanfei River, China	Taihu Lake, China	Dongting Lake, China	Poyang Lake, China	Surface water, Northern Greece	Mohawk River, USA	Surface water, USA	Urban rivers, Brazil
TPhP	0.12 (ND-4.86)	5.21 (0.87-11.68)	1.0 (0.7-4.2)	3.98 (0.09-8.41)	3.98 (0.53–13.2)	2.76 (ND-79.5)	11.0 (ND-27.8)	7.44 (2.39-128.55)	72.6 (40–258)	ND (ND-85.9)	6.9 (ND-36.5)	ND (ND–0.059)
TPrP	-	-	0.70 (0.3-12.6)	ND (ND-1.79)	-	2.11 (ND-10.9)	ND	0.11 (ND-0.58)	-	-	-	-
TMP	0.04 (ND-1.22)	4.72 (0.52-12.45)	-	1.88 (ND-20.4)	0.27 (ND-7.9)	0.13 (ND-1.55)	0.475 (ND-0.759)	0.72 (ND-126.61)	-	1.05 (ND-4.29)	-	-
TEP	26.2 (2.49-2405)	364.82 (34.22-1352.56)	-	-	753 (344–4200)	-	-	588.36 (172.72-2582.89)	58.5 (ND-134)	16.5 (ND-850)	4.77 (ND-24.8)	-
TDCPP	2.09 (ND-31.8)	35.21 (18.54-106.27)	6.6 (1.2-11.6)	1.50 (ND-6.35)	51.4 (2.10–97.5)	27.9 (9.74–682)	3.46 (ND–6.59)	727.91 (123.09-4095.81)	4.2 (ND-14)	68 (26.7–204)	21.1 (ND-86.7)	ND (ND-0.48)
TCEP	17.9 (0.40-1182)	34.65 (195.50-771.33)	34.65 (39.3-414.9)	8.53 (ND-30.9)	140.3 (5.20–750)	208.5 (31.6–1870)	44.5 (16.7–44.5)	19.80 (5.47-57.74)	76 (18–163)	26.9 (9.93–248)	14.6 (ND-79.5)	ND (ND–0.34)
TnBP	6.37 (0.46-172)	9.54 (41.21-82.15)	30.3 (13.6-179.0)	3.05 (0.13-8.89)	12.7 (2.00–65.3)	9.58 (3.61–334)	12.5 (ND–23.4)	10.26 (0.93–70.72)	10.26 (15–374)	4.41 (ND–358)	1.2 (ND–5.13)	ND (ND–0.281)
TBOEP	2.33 (0.34-34.5)	15.34 (6.24-135.92)	-	3.43 (ND-50.2)	18.1 (0.53–113)	33.75 (5.08–259)	ND	0.84 (ND–4.08)	458 (ND–997)	33.2 (1.22–1870)	66.2 (2.53–366)	ND (ND–3.6)
TCPP	34.6 (ND-649)	401.67 (82.37-973.93)	84.4 (37.8-260.9)	20.0 (ND-379)	178 (10.5–293)	434 (59.7–12300)	43.8 (27.3–106)	5.13 (ND–101.72)	92.5 (59–208)	427 (1.05–23300)	74.6 (3.3–214)	ND (ND–0.34)
Reference	[25]	[26]	[27]	[28]	[29]	[30]	[31]	[32]	[33]	[34]	[35]	[36]

Table S11. The chemical composition (organic contaminants) of the main industrial effluent wastewater

	PFASs (ng/L)	OPFRs (ng/L)	Pesticides (ng/L)	Antibiotics (ng/L)	Antimicrobials (ng/L)	PAEs (ng/L)
Effluent wastewater	36.41-926.93	33.95-2427.21	39.19-160.78	5.38-139.25	56.58-187.70	67.9-3184.33

Data source: P1–P6 effluent wastewater sample measurement.

Table S12. Spearman rank order correlation coefficients of PFASs in the water samples.

	PFOA	PFOS	8:2FTUCA	6:2FTS	8:2FTS	PFBA	PFBS	PFHpA	PFDA	PFHxA	PFHxS	PFNA	FOSA	PFPeA	ADONA	TFMS	TFA
PFOS	0.415**																
8:2 FTUCA	0.150	-0.053															
6:2 FTS	0.215	0.226	0.157														
8:2 FTS	0.142	0.126	0.137	-0.126													
PFBA	0.705***	0.529***	0.054	0.378*	0.133												
PFBS	0.240	0.022	0.314*	0.142	0.079	0.203											
PFHpA	0.811***	0.489**	0.083	0.175	0.306*	0.739***	0.252										
PFDA	0.645***	0.539***	-0.026	0.310*	0.218	0.580***	0.296	0.692***									
PFHxA	0.531***	-0.003	-0.006	-0.015	0.071	0.504***	0.203	0.442**	0.166								
PFHxS	0.174	0.515***	-0.093	0.281	0.100	0.256	-0.228	0.198	0.340*	-0.043							
PFNA	0.706***	0.393*	0.246	0.392*	0.221	0.555***	0.453*	0.699***	0.757***	0.187	0.281						
FOSA	-0.002	0.233	-0.072	0.254	-0.191	0.341*	-0.038	0.025	-0.032	0.165	-0.025	-0.056					
PFPeA	0.624***	0.351*	0.165	0.287	0.166	0.796***	0.319*	0.692***	0.397**	0.468**	-0.102	0.382*	0.376*				
ADONA	-0.122	-0.049	0.062	-0.042	-0.090	-0.084	-0.115	-0.185	-0.061	0.048	-0.200	-0.160	0.093	-0.067			
TFMS	0.067	-0.014	-0.173	0.051	-0.170	0.299	0.083	-0.001	-0.088	0.513***	0.020	-0.207	0.417**	0.284	-0.055		
TFA	0.159	0.390*	-0.171	0.047	-0.055	0.483**	0.151	0.208	0.164	0.125	0.189	0.010	0.287	0.362*	-0.095	0.507***	
HFPO-DA	0.354*	0.702***	-0.012	0.315*	0.093	0.715***	0.161	0.564***	0.408**	0.150	0.246	0.295	0.307*	0.614***	-0.193	0.258	0.520***

***The relationship is significant at $p < 0.001$; **the relationship is significant at $p < 0.01$; *the relationship is significant at $p < 0.05$; significant differences were compared using the nonparametric Spearman correlation analysis.

Table S13. Spearman rank order correlation coefficients of OPFRs in the water samples.

	TPhP	TPrP	TMP	TEP	TDCPP	TCEP	TnBP	TBOEP
TPrP	-0.045							
TMP	-0.053	0.128						
TEP	0.388*	-0.013	-0.040					
TDCPP	0.083	0.287	-0.131	-0.005*				
TCEP	0.428**	0.185	0.122	0.329	0.448**			
TnBP	0.243	-0.038	0.249	0.151	0.121	0.455**		
TBOEP	0.140	0.104	0.020	0.355*	0.168	0.403**	0.058	
TCPP	-0.010	0.121	0.4164**	0.245	-0.181	0.311*	0.243	0.213

***The relationship is significant at $p < 0.001$; **the relationship is significant at $p < 0.01$; *the relationship is significant at $p < 0.05$; significant differences were compared using the nonparametric Spearman correlation analysis.

Table S14. Annual emission flux (F: tons/year) of the PFASs and OPFRs discharged into the upper Yangtze River.

Period	Compound	Jinsha River	Min River	Tuo River	Jialing River	Wu River	Yangtze River
	PFOA	0.00	0.07	0.56	0.02	0.05	0.89
	PFOS	0.00	0.00	0.03	0.00	0.00	0.00
	8:2 FTUCA	0.00	0.03	0.04	0.22	0.01	0.22
	6:2 FTS	1.42	1.06	0.22	1.09	0.82	5.84
	8:2 FTS	0.12	0.03	0.05	0.07	0.03	0.21
	PFBA	0.08	0.11	0.07	0.06	0.14	0.48
	PFBS	0.00	0.12	0.05	0.14	0.00	0.04
	PFHpA	0.00	0.00	0.05	0.00	0.02	0.06
	PFDA	0.00	0.01	0.01	0.00	0.02	0.02
	PFHxA	0.10	0.77	0.05	0.01	0.02	1.31
	PFHxS	0.00	0.00	0.00	0.00	0.00	0.00
	PFNA	0.00	0.03	0.03	0.03	0.03	0.08
2022	FOSA	0.00	0.00	0.00	0.00	0.00	0.01
	PFPeA	0.00	0.00	0.03	0.00	0.04	0.03
	ADONA	0.00	0.02	0.00	0.00	0.02	0.02
	TFMS	0.01	7.62	0.06	0.34	0.00	41.72
	TFA	0.00	0.02	0.00	0.00	0.00	0.00
	HFPO-DA	0.00	0.00	0.01	0.00	0.00	0.00
	∑PFASs	1.73	9.88	1.27	1.97	1.20	50.93
	TPhP	0.63	0.18	0.01	0.02	0.15	0.15
	TPrP	0.00	0.08	0.01	0.00	0.00	0.29
	TMP	0.19	0.11	0.03	0.10	0.06	0.56
	TEP	0.56	0.13	0.00	0.12	0.03	1.28
	TDCPP	0.08	0.09	0.03	0.03	0.15	0.40
	TCEP	0.12	0.26	0.08	0.00	0.78	0.61

	TnBP	0.09	0.98	0.10	0.15	0.29	1.55
	TBOEP	0.00	0.01	0.00	0.13	0.01	0.34
	TCPP	3.18	3.16	0.35	2.65	0.38	12.67
	∑OPFRs	4.84	5.00	0.61	3.19	1.84	17.85
	PFOA	0.00	0.16	0.44	0.02	0.04	0.93
	PFOS	0.00	0.00	0.02	0.00	0.06	0.09
	8:2 FTUCA	0.07	0.03	0.03	0.03	0.01	0.31
	6:2 FTS	1.86	1.44	0.21	1.20	0.50	6.31
	8:2 FTS	0.11	0.05	0.01	0.03	0.00	0.30
	PFBA	0.23	0.21	0.10	0.06	0.04	1.17
	PFBS	0.00	0.42	0.18	0.00	0.00	0.67
	PFHpA	0.00	0.02	0.02	0.00	0.00	0.18
	PFDA	0.00	0.01	0.01	0.00	0.00	0.05
	PFHxA	0.00	0.74	0.05	0.00	0.00	1.47
	PFHxS	0.00	0.00	0.00	0.00	0.00	1.98
	PFNA	0.00	0.05	0.01	0.02	0.00	0.11
	FOSA	0.00	0.01	0.00	0.00	0.00	0.08
	PFPeA	0.00	0.07	0.05	0.00	0.00	0.63
2023	ADONA	0.09	0.03	0.00	0.02	0.04	0.08
	TFMS	0.43	30.81	4.45	0.73	0.66	121.04
	TFA	0.00	0.04	0.07	0.00	0.29	3.48
	HFPO-DA	0.00	0.00	0.02	0.00	0.00	0.29
	∑PFASs	2.79	34.08	5.70	2.10	1.64	139.16
	TPhP	0.69	0.04	0.00	0.02	0.08	0.14
	TPrP	0.00	0.72	0.03	3.14	0.05	4.21
	TMP	0.18	0.14	0.03	0.10	0.05	0.54
	TEP	0.84	0.08	0.02	0.20	0.18	0.95
	TDCPP	0.22	0.24	0.03	0.05	0.03	0.42
	TCEP	0.33	0.69	0.08	0.09	0.02	0.50
	TnBP	1.38	1.10	0.25	0.14	0.12	1.17
	TBOEP	0.00	0.14	0.05	0.05	0.00	0.36
	TCPP	0.00	2.96	0.29	3.45	0.00	12.20
	∑OPFRs	3.64	6.12	0.77	7.23	0.53	20.49

Table S15. Risk quotients (RQs) of PFASs and OPFRs for the aquatic organisms in the upper Yangtze River.

City	Sampling point	River name	PFAS RQs	OPFR RQs
2022				
Leshan	D1	Min	0.0112	0.0145
Yibin	D2	Jinsha	0.0002	0.0260
	D3	Min	0.0035	0.0275
	D4	Yangtze	0.0047	0.0019
Luzhou	D5	Yangtze	0.0094	0.0022
	D6	Tuo	0.0625	0.0177
	D7	Yangtze	0.0002	0.0227
Chongqing	D8	Jialing	0.0040	0.0033
	D9	Jialing	0.0029	0.0074
	D10	Yangtze	0.0136	0.0032
	D11	Yangtze	0.0095	0.0062
	D12	Wu	0.0079	0.0377
	D13	Yangtze	0.0094	0.0142
Leshan	P1	Min	0.0101	0.0602
	P2	Min	0.0333	0.4856
Yibin	P3	Yangtze	0.0325	0.0208
Luzhou	P4	Yangtze	0.0138	0.0030
Chongqing	P5	Jialing	0.0049	0.0021
	P6	Yangtze	0.0437	0.0730
2022				
Leshan	D1	Min	0.0264	0.0422
Yibin	D2	Jinsha	0.0004	0.0270
	D3	Min	0.0092	0.0099
	D4	Yangtze	0.0079	0.0426
Luzhou	D5	Yangtze	0.0176	0.0153
	D6	Tuo	0.0528	0.0149
	D7	Yangtze	0.0141	0.0045
Chongqing	D8	Jialing	0.0015	0.0943
	D9	Jialing	0.0018	0.0200
	D10	Yangtze	0.0057	0.0056
	D11	Yangtze	0.0122	0.1033
	D12	Wu	0.0023	0.0133
	D13	Yangtze	0.0046	0.0068
	D14	Yangtze	0.0198	0.0052
	D15	Yangtze	0.0617	0.0096
	D16	Yangtze	0.0322	0.0076
	D17	Yangtze	0.0160	0.0029
Leshan	P1	Min	0.0018	0.1001
	P2	Min	0.0197	1.2535
Yibin	P3	Yangtze	0.0314	0.0178
Luzhou	P4	Yangtze	0.0164	0.0067
Chongqing	P5	Jialing	0.0099	0.1776
	P6	Yangtze	0.0069	0.0519

Table S16. Non-carcinogenic risk (HI values) and carcinogenic risk (CR) of PFASs and OPFRs in the upper Yangtze River.

Compound	Non-carcinogenic risk (HI)								Carcinogenic risk (CR)							
	Infant		Child		Teenage		Adult		Infant		Child		Teenage		Adult	
	Median	Max.	Median	Max.	Median	Max.	Median	Max.	Median	Max.	Median	Max.	Median	Max.	Median	Max.
2022																
PFOA	1.39E-03	4.11E-02	4.97E-03	1.47E-01	3.18E-03	9.45E-02	3.01E-03	2.75E-02	1.77E-10	5.24E-09	3.91E-10	1.16E-08	7.52E-10	2.23E-08	3.61E-09	1.07E-07
PFOS	7.54E-03	7.54E-03	8.34E-03	8.34E-03	5.34E-03	5.34E-03	5.05E-03	6.57E-02	3.54E-10	3.54E-10	7.84E-10	7.84E-10	1.51E-09	1.51E-09	7.24E-09	7.24E-09
8:2 FTUCA	5.18E-06	2.41E-05	5.73E-06	2.66E-05	3.67E-06	1.71E-05	3.47E-06	1.16E-05	1.21E-10	5.63E-10	2.68E-10	1.25E-09	5.15E-10	2.39E-09	2.48E-09	1.15E-08
8:2 FTS	3.66E-06	2.24E-05	4.04E-06	2.47E-05	2.59E-06	1.59E-05	2.45E-06	1.09E-05	9.92E-11	6.07E-10	2.20E-10	1.34E-09	4.22E-10	2.58E-09	2.03E-09	1.24E-08
PFBA	3.61E-06	1.46E-05	3.99E-06	1.61E-05	2.56E-06	1.03E-05	2.42E-06	7.66E-06	6.58E-10	2.66E-09	1.46E-09	5.88E-09	2.80E-09	1.13E-08	1.34E-08	5.43E-08
PFBS	5.68E-06	1.04E-05	6.28E-06	1.15E-05	4.03E-06	7.39E-06	3.80E-06	1.54E-05	1.04E-09	1.90E-09	2.29E-09	4.21E-09	4.41E-09	8.09E-09	2.12E-08	3.89E-08
PFHpA	2.38E-06	2.01E-05	2.64E-06	2.22E-05	1.69E-06	1.42E-05	1.60E-06	1.52E-05	6.44E-11	5.43E-10	1.43E-10	1.20E-09	2.74E-10	2.31E-09	1.32E-09	1.11E-08
PFDA	1.94E-06	6.64E-06	2.15E-06	7.34E-06	1.37E-06	4.71E-06	1.30E-06	9.51E-06	5.78E-11	1.98E-10	1.28E-10	4.38E-10	2.46E-10	8.41E-10	1.18E-09	4.04E-09
PFHxA	1.87E-05	5.38E-05	2.07E-05	5.95E-05	1.32E-05	3.81E-05	1.25E-05	3.90E-05	7.03E-10	2.02E-09	1.55E-09	4.47E-09	2.99E-09	8.60E-09	1.44E-08	4.13E-08
PFNA	3.32E-06	1.35E-05	3.68E-06	1.50E-05	2.36E-06	9.60E-06	2.23E-06	1.10E-05	7.31E-11	2.98E-10	1.62E-10	6.59E-10	3.11E-10	1.27E-09	1.49E-09	6.09E-09
FOSA	2.73E-04	2.73E-04	3.01E-04	3.01E-04	1.93E-04	1.93E-04	1.83E-04	2.37E-03	1.28E-11	1.28E-11	2.83E-11	2.83E-11	5.45E-11	5.45E-11	2.62E-10	2.62E-10
PFPeA	2.57E-06	1.14E-05	2.84E-06	1.26E-05	1.82E-06	8.07E-06	1.72E-06	1.46E-05	1.25E-10	5.55E-10	2.77E-10	1.23E-09	5.32E-10	2.36E-09	2.56E-09	1.13E-08
ADONA	2.60E-05	2.67E-05	2.87E-05	2.96E-05	1.84E-05	1.90E-05	1.74E-05	8.74E-05	6.16E-10	6.34E-10	1.36E-09	1.40E-09	2.62E-09	2.70E-09	1.26E-08	1.29E-08
TFMS	2.24E-05	1.82E-04	2.48E-05	2.01E-04	1.59E-05	1.29E-04	1.50E-05	7.14E-05	1.26E-09	1.02E-08	2.78E-09	2.25E-08	5.34E-09	4.33E-08	2.56E-08	2.08E-07
TFA	1.43E-06	1.43E-06	1.58E-06	1.58E-06	1.02E-06	1.02E-06	9.59E-07	1.25E-05	7.25E-11	7.25E-11	1.60E-10	1.60E-10	3.08E-10	3.08E-10	1.48E-09	1.48E-09
HFPO-DA	1.76E-02	1.76E-02	1.95E-02	1.95E-02	1.25E-02	1.25E-02	1.18E-02	1.54E-01	-	-	-	-	-	-	-	-
∑PFASs	2.69E-02	6.70E-02	3.32E-02	1.76E-01	2.13E-02	1.13E-01	2.01E-02	2.50E-01	5.42E-09	2.58E-08	1.20E-08	5.72E-08	2.31E-08	1.10E-07	1.11E-07	5.28E-07
TPhP	1.19E-06	3.89E-06	1.31E-06	4.30E-06	8.41E-07	2.75E-06	7.94E-07	2.80E-06	4.59E-10	1.50E-09	1.90E-09	6.23E-09	3.45E-09	1.13E-08	3.66E-09	1.20E-08
TPrP	1.36E-05	4.82E-05	1.50E-05	5.34E-05	9.62E-06	3.42E-05	9.09E-06	5.03E-05	1.50E-09	5.33E-09	1.48E-10	5.25E-10	2.68E-10	9.54E-10	2.84E-10	1.01E-09
TMP	9.37E-06	1.37E-05	1.04E-05	1.52E-05	6.64E-06	9.72E-06	6.27E-06	9.09E-06	4.50E-11	6.58E-11	1.05E-10	1.54E-10	1.91E-10	2.79E-10	2.02E-10	2.95E-10
TEP	8.27E-07	5.03E-06	9.15E-07	5.56E-06	5.86E-07	3.57E-06	5.54E-07	2.33E-06	2.32E-10	1.41E-09	4.97E-10	3.02E-09	9.03E-10	5.49E-09	9.55E-10	5.81E-09
TDCPP	2.06E-06	1.01E-05	2.28E-06	1.12E-05	1.46E-06	7.15E-06	1.38E-06	3.77E-06	3.52E-11	1.73E-10	7.15E-11	3.50E-10	1.30E-10	6.36E-10	1.37E-10	6.73E-10
TCEP	1.87E-06	7.93E-06	2.07E-06	8.77E-06	1.33E-06	5.62E-06	1.25E-06	6.68E-06	3.82E-10	1.62E-09	1.12E-09	4.76E-09	2.04E-09	8.65E-09	2.16E-09	9.16E-09
TnBP	2.57E-05	8.86E-05	2.84E-05	9.80E-05	1.82E-05	6.28E-05	1.72E-05	4.62E-05	5.79E-11	2.00E-10	1.29E-10	4.47E-10	2.35E-10	8.11E-10	2.49E-10	8.59E-10
TBOEP	8.85E-06	2.32E-05	9.79E-06	2.56E-05	6.27E-06	1.64E-05	5.93E-06	4.28E-05	1.92E-10	5.04E-10	3.11E-10	8.15E-10	5.66E-10	1.48E-09	5.99E-10	1.57E-09
TCPP	6.97E-04	1.14E-03	7.71E-04	1.26E-03	4.94E-04	8.07E-04	4.67E-04	8.03E-04	8.89E-09	1.45E-08	2.17E-08	3.55E-08	3.95E-08	6.45E-08	4.18E-08	6.82E-08

∑OPFRs	7.61E-04	1.34E-03	8.41E-04	1.48E-03	5.39E-04	9.50E-04	5.10E-04	9.67E-04	1.18E-08	2.53E-08	2.60E-08	5.18E-08	4.73E-08	9.41E-08	5.00E-08	9.96E-08
2022																
PFOA	5.50E-03	1.01E-01	6.09E-03	1.11E-01	3.90E-03	7.13E-02	3.69E-03	4.14E-02	2.16E-10	3.96E-09	4.79E-10	8.75E-09	8.70E-10	1.59E-08	9.21E-10	1.68E-08
PFOS	5.02E-03	5.13E-03	5.55E-03	5.68E-03	3.55E-03	3.64E-03	3.36E-03	2.92E-02	2.36E-10	2.41E-10	5.21E-10	5.34E-10	9.47E-10	9.70E-10	1.00E-09	1.03E-09
8:2 FTUCA	2.73E-06	1.52E-05	3.02E-06	1.68E-05	1.94E-06	1.08E-05	1.83E-06	8.92E-06	6.39E-11	3.56E-10	1.41E-10	7.87E-10	2.57E-10	1.43E-09	2.72E-10	1.51E-09
8:2 FTS	3.57E-06	5.54E-06	3.95E-06	6.12E-06	2.53E-06	3.92E-06	2.39E-06	5.31E-06	9.68E-11	1.50E-10	2.14E-10	3.32E-10	3.89E-10	6.04E-10	4.12E-10	6.39E-10
PFBA	4.09E-06	2.03E-05	4.53E-06	2.24E-05	2.90E-06	1.44E-05	2.74E-06	1.11E-05	7.46E-10	3.69E-09	1.65E-09	8.17E-09	3.00E-09	1.48E-08	3.17E-09	1.57E-08
PFBS	4.72E-06	3.77E-05	5.22E-06	4.16E-05	3.35E-06	2.67E-05	3.16E-06	2.89E-05	8.62E-10	6.87E-09	1.91E-09	1.52E-08	3.46E-09	2.76E-08	3.66E-09	2.92E-08
PFHpA	5.46E-06	8.66E-06	6.04E-06	9.58E-06	3.87E-06	6.14E-06	3.66E-06	3.23E-05	1.48E-10	2.34E-10	3.27E-10	5.18E-10	5.93E-10	9.40E-10	6.28E-10	9.95E-10
PFDA	1.52E-06	3.62E-06	1.68E-06	4.01E-06	1.07E-06	2.57E-06	1.01E-06	9.40E-06	4.52E-11	1.08E-10	9.99E-11	2.39E-10	1.81E-10	4.34E-10	1.92E-10	4.59E-10
PFHxA	4.12E-06	9.44E-06	4.56E-06	1.04E-05	2.92E-06	6.69E-06	2.76E-06	7.11E-06	1.55E-10	3.55E-10	3.43E-10	7.85E-10	6.23E-10	1.43E-09	6.60E-10	1.51E-09
PFNA	1.46E-06	6.03E-06	1.62E-06	6.67E-06	1.04E-06	4.27E-06	9.78E-07	3.85E-06	3.21E-11	1.33E-10	7.11E-11	2.93E-10	1.29E-10	5.33E-10	1.37E-10	5.64E-10
FOSA	3.33E-04	1.65E-03	3.68E-04	1.82E-03	2.36E-04	1.17E-03	2.23E-04	1.26E-03	1.56E-11	7.74E-11	3.46E-11	1.71E-10	6.28E-11	3.11E-10	6.65E-11	3.29E-10
PFPeA	2.36E-06	1.64E-05	2.62E-06	1.81E-05	1.68E-06	1.16E-05	1.58E-06	1.21E-05	1.15E-10	7.99E-10	2.55E-10	1.77E-09	4.63E-10	3.21E-09	4.90E-10	3.40E-09
ADONA	4.03E-06	7.12E-06	4.46E-06	7.88E-06	2.86E-06	5.05E-06	2.70E-06	7.02E-06	9.56E-11	1.69E-10	2.11E-10	3.73E-10	3.84E-10	6.79E-10	4.06E-10	7.18E-10
TFMS	7.76E-05	3.73E-04	8.58E-05	4.12E-04	5.50E-05	2.64E-04	5.20E-05	1.57E-04	4.34E-09	2.08E-08	9.60E-09	4.61E-08	1.74E-08	8.38E-08	1.84E-08	8.86E-08
TFA	1.04E-05	2.29E-05	1.15E-05	2.53E-05	7.40E-06	1.62E-05	6.99E-06	3.34E-05	5.28E-10	1.16E-09	1.17E-09	2.56E-09	2.12E-09	4.66E-09	2.25E-09	4.93E-09
HFPO-DA	3.68E-02	3.68E-02	4.07E-02	4.07E-02	2.61E-02	2.61E-02	2.46E-02	4.19E-01	-	-	-	-	-	-	-	-
∑PFASs	4.77E-02	1.45E-01	5.28E-02	1.60E-01	3.38E-02	1.03E-01	3.20E-02	4.91E-01	7.69E-09	3.91E-08	1.70E-08	8.66E-08	3.09E-08	1.57E-07	3.27E-08	1.66E-07
TPhP	7.87E-07	3.80E-06	8.71E-07	4.20E-06	5.58E-07	2.69E-06	5.27E-07	3.40E-06	5.71E-10	2.75E-09	1.26E-09	6.09E-09	2.29E-09	1.11E-08	2.43E-09	1.17E-08
TPrP	1.12E-04	1.21E-03	1.24E-04	1.34E-03	7.95E-05	8.56E-04	7.52E-05	4.64E-04	5.52E-10	5.95E-09	1.22E-09	1.32E-08	2.22E-09	2.39E-08	2.35E-09	2.53E-08
TMP	8.52E-06	1.23E-05	9.43E-06	1.36E-05	6.04E-06	8.70E-06	5.71E-06	7.81E-06	4.32E-11	6.21E-11	9.55E-11	1.37E-10	1.73E-10	2.50E-10	1.84E-10	2.64E-10
TEP	8.40E-07	6.36E-06	9.29E-07	7.03E-06	5.95E-07	4.51E-06	5.63E-07	2.89E-06	2.28E-10	1.73E-09	5.04E-10	3.82E-09	9.17E-10	6.94E-09	9.70E-10	7.34E-09
TDCPP	2.70E-06	1.31E-05	2.98E-06	1.45E-05	1.91E-06	9.28E-06	1.81E-06	5.88E-06	4.23E-11	2.05E-10	9.36E-11	4.54E-10	1.70E-10	8.26E-10	1.80E-10	8.74E-10
TCEP	7.51E-07	6.37E-06	8.31E-07	7.04E-06	5.32E-07	4.51E-06	5.03E-07	2.87E-06	2.04E-10	1.73E-09	4.51E-10	3.82E-09	8.20E-10	6.95E-09	8.67E-10	7.35E-09
TnBP	1.85E-05	1.11E-04	2.04E-05	1.23E-04	1.31E-05	7.90E-05	1.24E-05	4.22E-05	4.21E-11	2.54E-10	9.30E-11	5.62E-10	1.69E-10	1.02E-09	1.79E-10	1.08E-09
TBOEP	5.90E-06	2.72E-05	6.52E-06	3.01E-05	4.18E-06	1.93E-05	3.95E-06	2.45E-05	9.38E-11	4.33E-10	2.08E-10	9.58E-10	3.77E-10	1.74E-09	3.99E-10	1.84E-09
TCPP	6.08E-04	1.30E-03	6.72E-04	1.44E-03	4.31E-04	9.21E-04	4.07E-04	8.81E-04	8.56E-09	1.83E-08	1.89E-08	4.05E-08	3.44E-08	7.35E-08	3.64E-08	7.78E-08
∑OPFRs	7.58E-04	2.69E-03	8.38E-04	2.97E-03	5.37E-04	1.90E-03	5.08E-04	1.43E-03	1.03E-08	3.14E-08	2.29E-08	6.95E-08	4.15E-08	1.26E-07	4.39E-08	1.34E-07

—: no estimated data were available.

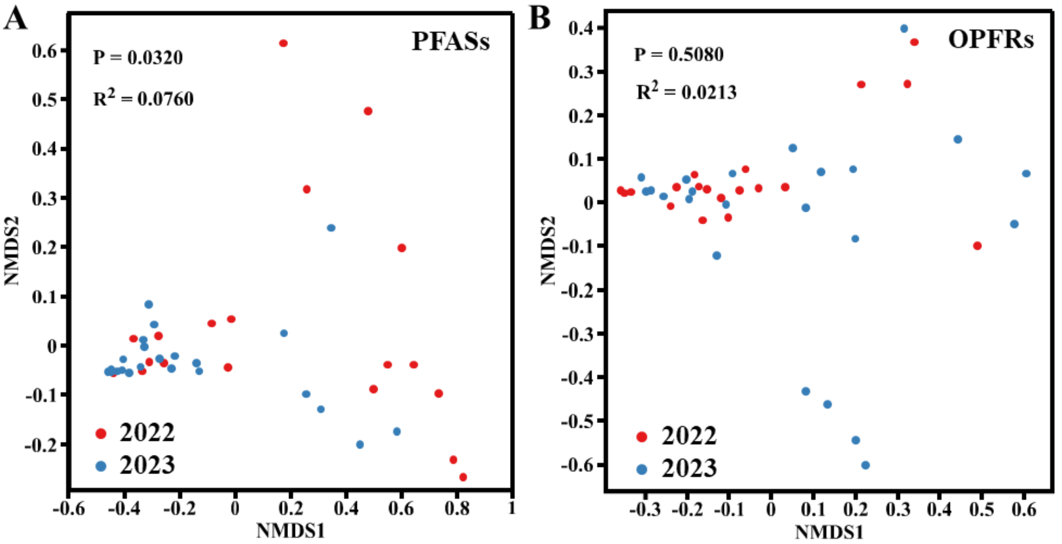

Figure S1. NMDS analyses of PFAS (A) and OPFR (B) concentrations in the water samples in 2022 and 2023.

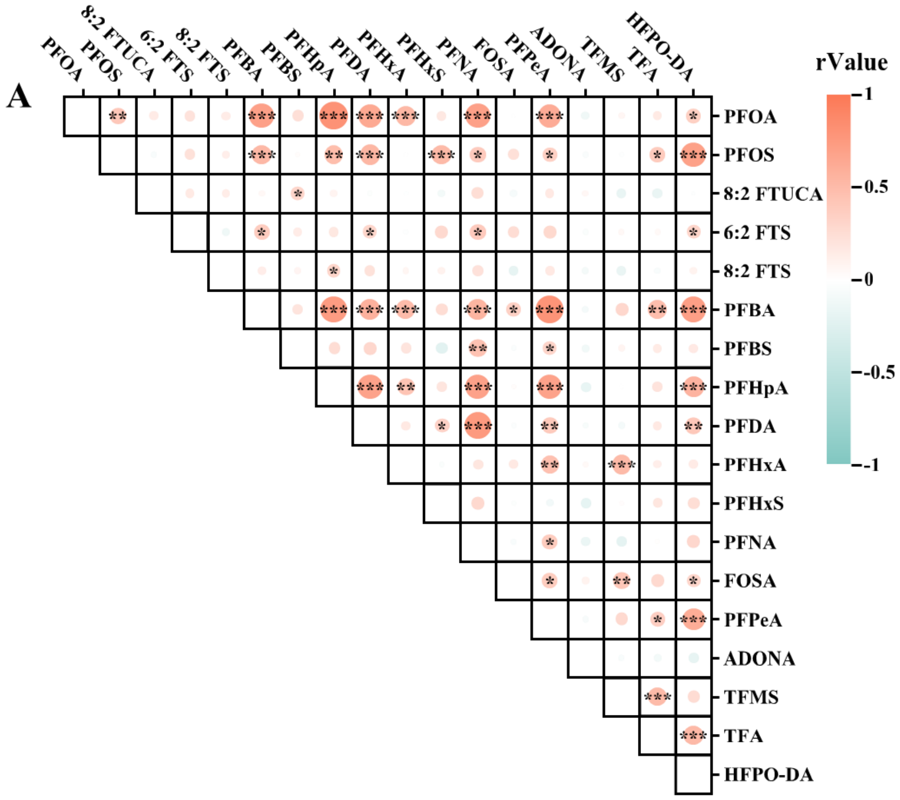

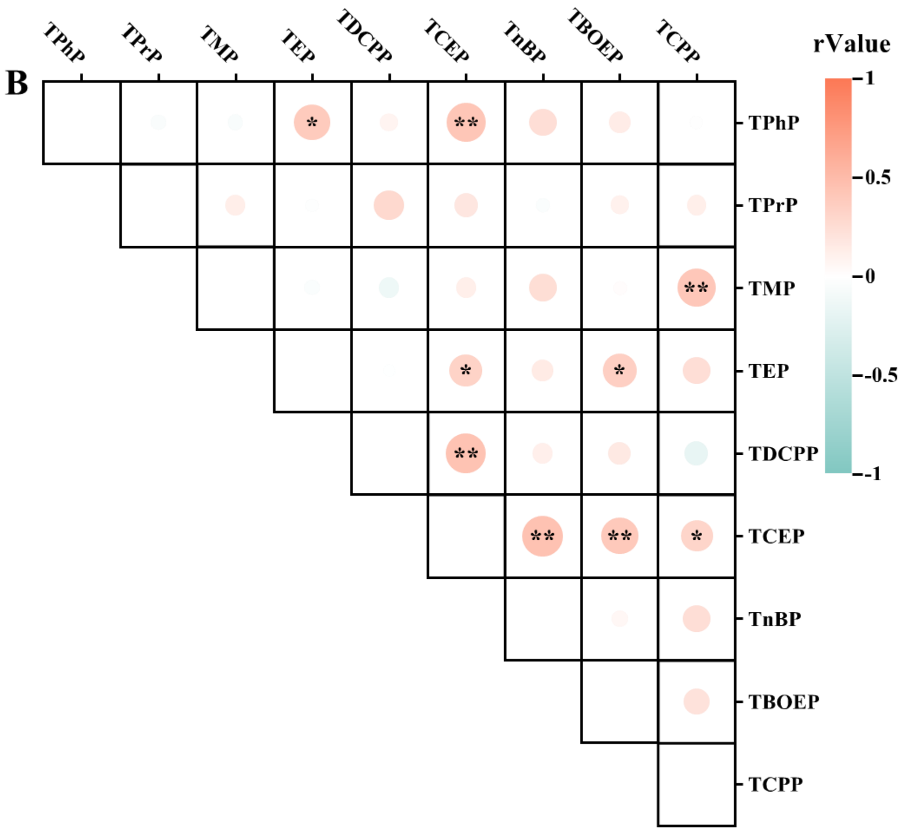

Figure S2. The calculated correlation between each concentration of PFASs (A) and OPFRs (B) based on the non-parametric measure of Spearman correlation.

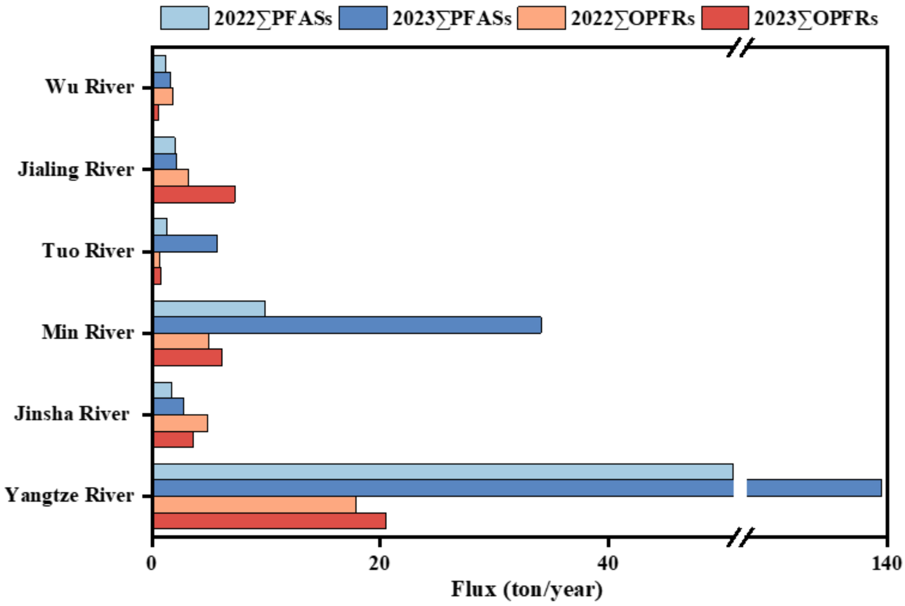

Figure S3. Annual emission flux (F: tons/year) of PFASs and OPFRs.

Reference：

1. Li, J.; Ai, Y.; Hu, J.; Xu, N.; Song, R.; Zhu, Y.; Sun, W.; Ni, J. Polyfluoroalkyl substances in Danjiangkou Reservoir, China: occurrence, composition, and source appointment. *Sci. Total Environ.* **2020**, *725*, 138352, doi:https://doi.org/10.1016/j.scitotenv.2020.138352.
2. Hoke, R.A.; Bouchelle, L.D.; Ferrell, B.D.; Buck, R.C. Comparative acute freshwater hazard assessment and preliminary PNEC development for eight fluorinated acids. *Chemosphere* **2012**, *87*, 725-733, doi:https://doi.org/10.1016/j.chemosphere.2011.12.066.
3. Hu, J.; Lyu, Y.; Chen, H.; Cai, L.; Li, J.; Cao, X.; Sun, W. Integration of target, suspect, and nontarget screening with risk modeling for per-and polyfluoroalkyl substances prioritization in surface waters. *Water Res.* **2023**, *233*, 119735, doi:https://doi.org/10.1016/j.watres.2023.119735.
4. Gebreab, K.Y.; Benetti, D.; Grosell, M.; Stieglitz, J.D.; Berry, J.P. Toxicity of perfluoroalkyl substances (PFAS) toward embryonic stages of mahi-mahi (Coryphaena hippurus). *Ecotoxicology* **2022**, *31*, 1057-1067, doi:https://doi.org/10.1007/s10646-022-02576-w.
5. Valsecchi, S.; Conti, D.; Crebelli, R.; Polesello, S.; Rusconi, M.; Mazzoni, M.; Preziosi, E.; Carere, M.; Lucentini, L.; Ferretti, E. Deriving environmental quality standards for perfluorooctanoic acid (PFOA) and related short chain perfluorinated alkyl acids. *J. Hazard. Mater.* **2017**, *323*, 84-98, doi:https://doi.org/10.1016/j.jhazmat.2016.04.055.
6. Ulhaq, M.; Carlsson, G.; Örn, S.; Norrgren, L. Comparison of developmental toxicity of seven perfluoroalkyl acids to zebrafish embryos. *Environ. Toxicol. Pharmacol.* **2013**, *36*, 423-426, doi:https://doi.org/10.1016/j.etap.2013.05.004.
7. Li, J.; Gao, Y.; Xu, N.; Li, B.; An, R.; Sun, W.; Borthwick, A.G.L.; Ni, J. Perfluoroalkyl substances in the Yangtze River: Changing exposure and its implications after operation of the Three Gorges Dam. *Water Res.* **2020**, *182*, 115933, doi:https://doi.org/10.1016/j.watres.2020.115933.
8. OECD. *Test No. 201: Freshwater Alga and Cyanobacteria, Growth Inhibition Test*; 2011. doi:https://doi.org/10.1787/9789264069923-en.
9. OECD. *Test No. 202: Daphnia sp. Acute Immobilisation Test*; 2004. doi:https://doi.org/10.1787/9789264069947-en.
10. OECD. *Test No. 203: Fish, Acute Toxicity Test*; 2019. doi:https://doi.org/10.1787/9789264069961-en.
11. Berends, A.G.; Boutonnet, J.C.; Rooij, C.G.D.; Thompson, R.S. Toxicity of trifluoroacetate to aquatic organisms. *Environmental Toxicology and Chemistry: An International Journal* **1999**, *18*, 1053-1059, doi:https://doi.org/10.1002/etc.5620180533.
12. Garavagno, M.d.l.A.; Holland, R.; Khan, M.A.H.; Orr-Ewing, A.J.; Shallcross, D.E. Trifluoroacetic Acid: Toxicity, Sources, Sinks and Future Prospects. *Sustainability* **2024**, *16*, 2382, doi:https://doi.org/10.3390/su16062382.
13. Leng, Y.; Xiao, H.; Li, Z.; Liu, Y.; Huang, K.; Wang, J. Occurrence and ecotoxicological risk assessment of perfluoroalkyl substances in water of lakes along the middle reach of Yangtze River, China. *Sci. Total Environ.* **2021**, *788*, 147765, doi:https://doi.org/10.1016/j.scitotenv.2021.147765.
14. Wang, G.; Shi, H.; Du, Z.; Chen, H.; Peng, J.; Gao, S. Bioaccumulation mechanism of organophosphate esters in adult zebrafish (Danio rerio). *Environ. Pollut.* **2017**, *229*, 177-187, doi:https://doi.org/10.1016/j.envpol.2017.05.075.
15. Zhao, J.; Guo, C.; Yang, Q.; Liu, W.; Zhang, H.; Luo, Y.; Zhang, Y.; Wang, L.; Chen, C.; Xu, J. Comprehensive monitoring and prioritizing for contaminants of emerging concern in the Upper Yangtze River, China: An integrated approach. *J. Hazard. Mater.* **2024**, *480*, 135835, doi:https://doi.org/10.1016/j.jhazmat.2024.135835.
16. Meng, P.; Sheppard, N.; Joseph, S.; Duckworth, O.W.; Higgins, C.P.; Knappe, D.R.U. Residential Garden Produce Harvested Near a Fluorochemical Manufacturer in North Carolina Can Be An Important

Fluoroether Exposure Pathway. *J. Agric. Food Chem.* **2024**, *72*, 26874-26883, doi:https://doi.org/10.1021/acs.jafc.4c06177.

17. Lian, M.; Lin, C.; Li, Y.; Hao, X.; Wang, A.; He, M.; Liu, X.; Ouyang, W. Distribution, partitioning, and health risk assessment of organophosphate esters in a major tributary of middle Yangtze River using Monte Carlo simulation. *Water Res.* **2022**, *219*, 118559, doi:https://doi.org/10.1016/j.watres.2022.118559.
18. Xing, L.; Tao, M.; Zhang, Q.; Kong, M.; Sun, J.; Jia, S.; Liu, C.-H. Occurrence, spatial distribution and risk assessment of organophosphate esters in surface water from the lower Yangtze River Basin. *Sci. Total Environ.* **2020**, *734*, 139380, doi:https://doi.org/10.1016/j.scitotenv.2020.139380.
19. Pan, Y.; Zhang, H.; Cui, Q.; Sheng, N.; Yeung, L.W.Y.; Sun, Y.; Guo, Y.; Dai, J. Worldwide Distribution of Novel Perfluoroether Carboxylic and Sulfonic Acids in Surface Water. *Environ. Sci. Technol.* **2018**, *52*, 7621-7629, doi:https://doi.org/10.1021/acs.est.8b00829.
20. Pan, C.; Ying, G.; Zhao, J.; Liu, Y.; Jiang, Y.; Zhang, Q. Spatiotemporal distribution and mass loadings of perfluoroalkyl substances in the Yangtze River of China. *Sci. Total Environ.* **2014**, *493*, 580-587, doi:https://doi.org/10.1016/j.scitotenv.2014.06.033.
21. An, W.; Duan, L.; Zhang, Y.; Wang, B.; Liu, C.S.; Wang, F.; Sui, Q.; Xu, D.; Yu, G. Occurrence, spatiotemporal distribution, seasonal and annual variation, and source apportionment of poly–and perfluoroalkyl substances (PFASs) in the northwest of Tai Lake Basin, China. *J. Hazard. Mater.* **2021**, *416*, 125784, doi:https://doi.org/10.1016/j.jhazmat.2021.125784.
22. Tang, A.; Zhang, X.; Li, R.; Tu, W.; Guo, H.; Zhang, Y.; Li, Z.; Liu, Y.; Mai, B. Spatiotemporal distribution, partitioning behavior and flux of per- and polyfluoroalkyl substances in surface water and sediment from Poyang Lake, China. *Chemosphere* **2022**, *295*, 133855, doi:https://doi.org/10.1016/j.chemosphere.2022.133855.
23. Neuwald, I.J.; Hu¨bner, D.; Wiegand, H.L.; Valkov, V.; Borchers, U.; No¨dler, K.; Scheurer, M.; Hale, S.E.; Arp, H.P.H.; Zahn, D. Ultra-short-chain PFASs in the sources of German drinking water: prevalent, overlooked, difficult to remove, and unregulated. *Environ. Sci. Technol.* **2022**, *56*, 6380-6390, doi:https://doi.org/10.1021/acs.est.1c07949.
24. Zhi, Y.; Lu, X.; Munoz, G.; Yeung, L.W.Y.; De Silva, A.O.; Hao, S.; He, H.; Jia, Y.; Higgins, C.P.; Zhang, C. Environmental Occurrence and Biotic Concentrations of Ultrashort-Chain Perfluoroalkyl Acids: Overlooked Global Organofluorine Contaminants. *Environ. Sci. Technol.* **2024**, doi:https://doi.org/10.1021/acs.est.4c04453.
25. Li, S.; Wan, Y.; Wang, Y.; He, Z.; Xu, S.; Xia, W. Occurrence, spatial variation, seasonal difference, and ecological risk assessment of organophosphate esters in the Yangtze River, China: From the upper to lower reaches. *Sci. Total Environ.* **2022**, *851*, 158021, doi:https://doi.org/10.1016/j.scitotenv.2022.158021.
26. Li, W.; Yuan, Y.; Wang, S.; Liu, X. Occurrence, spatiotemporal variation, and ecological risks of organophosphate esters in the water and sediment of the middle and lower streams of the Yellow River and its important tributaries. *J. Hazard. Mater.* **2023**, *443*, 130153, doi:https://doi.org/10.1016/j.jhazmat.2022.130153.
27. Sun, H.; Mi, W.; Li, X.; Wang, S.; Yan, J.; Zhang, G. Organophosphate ester in surface water of the Pearl River and South China Sea, China: Spatial variations and ecological risks. *Chemosphere* **2024**, 142559, doi:https://doi.org/10.1016/j.chemosphere.2024.142559.
28. Liu, Y.; Chen, L.; Li, H.; Song, Y.; Yang, Z.; Cui, Y. Occurrence of organophosphorus flame retardants in Xiangjiang River: Spatiotemporal variations, potential affecting factors, and source apportionment. *Chemosphere* **2024**, *355*, 141822, doi:https://doi.org/10.1016/j.chemosphere.2024.141822.
29. Li, Y.; Yao, C.; Zheng, Q.; Yang, W.; Niu, X.; Zhang, Y.; Lu, G. Occurrence and ecological implications of organophosphate triesters and diester degradation products in wastewater, river water, and tap water. *Environ. Pollut.* **2020**, *259*, 113810, doi:https://doi.org/10.1016/j.envpol.2019.113810.
30. Wang, X.; Zhu, L.; Zhong, W.; Yang, L. Partition and source identification of organophosphate esters in the

water and sediment of Taihu Lake, China. *J. Hazard. Mater.* **2018**, *360*, 43-50, doi:https://doi.org/10.1016/j.jhazmat.2018.07.082.

31. Xu, L.; Zhang, B.; Hu, Q.; Liu, Y.; Shang, T.; Zeng, X.; Yu, Z. Occurrence and spatio-seasonal distribution of organophosphate tri-and di-esters in surface water from Dongting Lake and their potential biological risk. *Environ. Pollut.* **2021**, *282*, 117031, doi:https://doi.org/10.1016/j.envpol.2021.117031.
32. Yan, Z.; Feng, C.; Xu, Y.; Wang, J.; Huang, N.; Jin, X.; Wu, F.; Bai, Y. Water temperature governs organophosphate ester dynamics in the aquatic food chain of Poyang Lake. *Environmental Science and Ecotechnology* **2024**, *21*, 100401, doi:https://doi.org/10.1016/j.ese.2024.100401.
33. Pantelaki, I.; Voutsa, D. Organophosphate esters in inland and coastal waters in northern Greece. *Sci. Total Environ.* **2021**, *800*, 149544, doi:https://doi.org/10.1016/j.scitotenv.2021.149544.
34. Li, W.; Wang, Y.; Kannan, K. Occurrence, distribution and human exposure to 20 organophosphate esters in air, soil, pine needles, river water, and dust samples collected around an airport in New York state, United States. *Environ. Int.* **2019**, *131*, 105054, doi:https://doi.org/10.1016/j.envint.2019.105054.
35. Kim, U.-J.; Kannan, K. Occurrence and Distribution of Organophosphate Flame Retardants/Plasticizers in Surface Waters, Tap Water, and Rainwater: Implications for Human Exposure. *Environ. Sci. Technol.* **2018**, *52*, 5625-5633, doi:https://doi.org/10.1021/acs.est.8b00727.
36. Cristale, J.; Oliveira Santos, I.; Umbuzeiro, G.d.A.; Fagnani, E. Occurrence and risk assessment of organophosphate esters in urban rivers from Piracicaba watershed (Brazil). *Environmental Science and Pollution Research* **2021**, *28*, 59244-59255, doi:https://doi.org/10.1007/s11356-020-10150-2.
